# Ultra‐Long‐Term Anti‐Inflammatory Polyphenol Capsule to Remodel the Microenvironment for Accelerating Osteoarthritis Healing by Single Dosage

**DOI:** 10.1002/advs.202407425

**Published:** 2024-11-18

**Authors:** Shaoyin Wei, Zeyu Shou, Dong Yang, Linxiao Sun, Yan Guo, Yang Wang, Xingjie Zan, Lianxin Li, Chunwu Zhang

**Affiliations:** ^1^ School of Ophthalmology and Optometry Eye Hospital School of Biomedical Engineering Wenzhou Medical University Wenzhou 325035 China; ^2^ Wenzhou Key Laboratory of Perioperative Medicine Wenzhou Institute University of Chinese Academy of Sciences Wenzhou 325001 China; ^3^ Department of Orthopedics Zhuji Affiliated Hospital of Wenzhou Medical University Shaoxing 311800 China; ^4^ Key Laboratory of Diagnosis and Treatment of Severe Hepato‐Pancreatic Diseases of Zhejiang Province The First Affiliated Hospital of Wenzhou Medical University Wenzhou 325000 China; ^5^ Hunan Provincial Key Laboratory of Advanced Materials for New Energy Storage and Conversion School of Materials Science and Engineering Hunan University of Science and Technology Xiangtan 411201 China; ^6^ Department of Orthopaedics Surgery Shandong Provincial Hospital Affiliated to Shandong First Medical University Jinan Shandong 250021 China; ^7^ The First Affiliated Hospital of Wenzhou Medical University Wenzhou 325000 China

**Keywords:** antioxidant, capsules, osteoarthritis, polyphenol, single‐dose cure

## Abstract

Osteoarthritis (OA) is a common chronic inflammatory disease that leads to disability and death. Existing therapeutic agents often require frequent use, which can lead to drug resistance and long‐term side effects. Polyphenols have anti‐inflammatory and antioxidant potential. However, they are limited by their short half‐life and low bioavailability. This work presents a novel pure polyphenol capsule for sustained release of polyphenols, which is self‐assembled via hydrophobic and hydrogen bonds. The capsule enhances cellular uptake, scavenges reactive oxygen and nitrogen species, reduces inflammatory markers, and remodels the OA microenvironment by inhibiting the p38 MAPK pathway. The capsule overcomes the limitations of short half‐life and low bioavailability of polyphenols and achieves single‐dose cure in mouse and dog OA models, providing an optimal therapeutic window for OA repair. Taking advantage of simple manufacturing, convenient administration, and pure polyphenol composition, these capsules show great potential for clinical treatment of osteoarthritis and chronic inflammatory diseases.

## Graphical abstract

A universal strategy to generate well‐dispersed and stable pure polyphenol capsules without metal ions available which achieved ultra‐slow release (2 months) and long‐lasting free radical scavenging (6 months) of proanthocyanidins, and the capsules showed promising ability to accelerate the healing of osteoarthritis by regulating multiple and common intracellular MAPK signaling pathway.

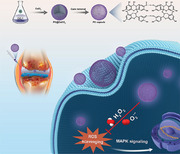

## 1 Introduction

Osteoarthritis (OA) represents a chronic inflammatory disease characterized by degeneration of articular cartilage and an inflammatory response in the surrounding tissues,^[^ ^1^ ^]^ leading to irreversible damage and high mortality rates,^[^ ^2^ ^]^ severely affecting the lives of patients and burdening the public health system. Excessive reactive oxygen species (ROS) and the inflammatory microenvironment play a central role in the pathogenesis of OA,^[^ ^3^ ^]^ with ROS directly attacking articular chondrocytes and activating inflammatory cells, leading to structural damage, release of inflammatory mediators, and destruction of articular cartilage.^[^ ^4^ ^]^ Osteoarthritis patients have insufficient antioxidant capacity to effectively scavenge excess ROS, leading to persistent oxidative stress and exacerbation of the condition.^[^ ^5^ ^]^

Clinically, small molecule drugs such as aspirin,^[^ ^6^ ^]^ ibuprofen,^[^ ^7^ ^]^ and naproxen,^[^ ^8^ ^]^ are commonly used to treat osteoarthritis by blocking pro‐inflammatory cytokines, proteases, and inflammatory signaling.^[^ ^9^ ^]^ However, these drugs have a short half‐life and require frequent dosing, which affects patient compliance, efficacy, and duration of treatment, and may lead to drug resistance and side effects with long‐term use.^[^ ^10^ ^]^ Although nano‐delivery systems can change the pharmacokinetics of drugs, they are still unable to meet the long‐term anti‐inflammatory needs of osteoarthritis.^[^ ^11^ ^]^ Therefore, new strategies are urgently needed to achieve long‐term sustained release of anti‐inflammatory drugs to effectively control osteoarthritis and reduce complications.^[^ ^12^ ^]^

Polyphenols are well known for their anti‐inflammatory and antioxidant properties and have been found in more than 8000 compounds. Polyphenols exert their anti‐inflammatory effects through multiple pathways, such as scavenging ROS,^[^ ^13^ ^]^ protecting cell membrane function and structure,^[^ ^14^ ^]^ and inhibiting enzyme activities associated with inflammation.^[^ ^15^ ^]^ Recent studies have shown that tea polyphenols modulate various intracellular signaling pathways such as MAPK (mitogen‐activated protein kinase), PI3K/Akt (phosphatidylinositol 3 kinase/protein kinase B), and NF‐κB (nuclear factor kappa‐light‐chain‐enhancer of activated B cells).^[^ ^16^ ^]^ However, it is difficult to avoid the limitations of free polyphenols, similar to those of small molecule drugs, when administered orally or intra‐articularly, resulting in poor therapeutic efficacy.

Polyphenols can interact with a wide range of molecules and can be incorporated into multiple delivery systems to improve stability and bioavailability. Despite advances in OA therapy, polyphenol delivery systems do not prolong drug metabolism and require multiple doses.^[^ ^17^ ^]^ The delivery of polyphenols requires the use of excipients, which can raise concerns about side effects and biosafety.^[^ ^17^, ^18^ ^]^ The key to meeting the long‐term needs of OA repair is to increase the cellular uptake and sustained release of polyphenols and to restore their biological activity. However, this is difficult to achieve with existing strategies. In addition, the interaction of polyphenols with encapsulation materials may affect polyphenol signaling pathways that are less involved in OA.

**Scheme 1:**
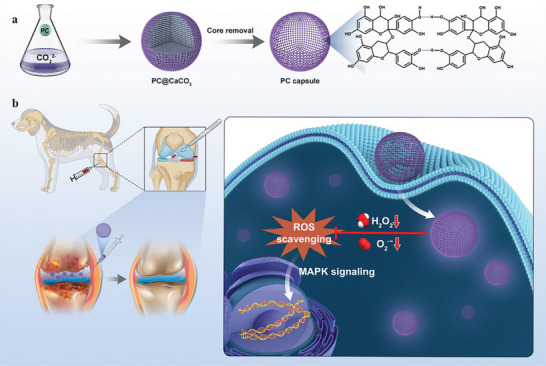
Schematic illustration of PC capsules in the treatment of ROS‐related diseases. a) PC capsules with broad‐spectrum and long‐term ROS scavenging ability are synthesized by a simple and green method. b) Due to the inhibition of the MAPK signaling pathway in vivo, PC capsules exhibit therapeutic effects against osteoarthritis.

Herein, we propose an efficient strategy to prepare proanthocyanidin (PC) capsules by a template‐based one‐step method without trapping substrates or metal ions (**Scheme** **1**a). It has been shown that such polyphenol capsules have good bio‐storage activity and can release polyphenols continuously for months. More importantly, the polyphenol capsules are efficiently transported across cell membranes and exerted a potent anti‐inflammatory effect in a variety of cellular models by modulating the MAPK signaling pathway (Scheme 1b), which is closely associated with inflammatory responses. This strategy is expected to improve the bioavailability of polyphenols and remodel the microenvironment around OA in mouse models and dog models after a single administration.

## 2 Results

### 2.1 Preparation and Characterization of PC Capsules

**Figure 1:**
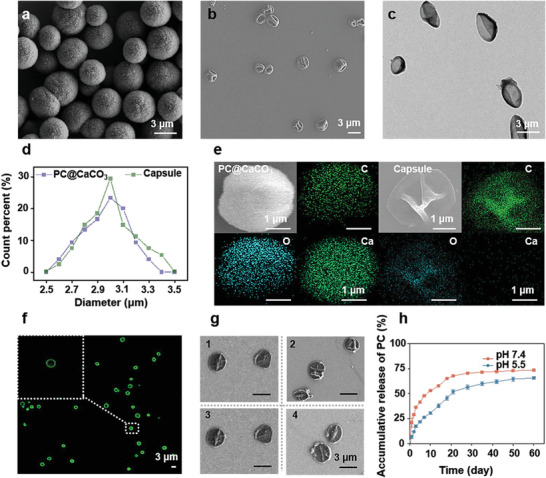
Preparation and characterization of PC capsules. a) SEM image of PC@CaCO_3_. b) SEM image of PC capsules. c) TEM image of PC capsule. d) Size distribution of PC@CaCO_3_ and capsules. e) EDS analysis of PC@CaCO_3_ and capsules. f) CLSM image of PC capsule. g) Stability of the capsules in 1) pure water, 2) PBS, 3) 0.9% NaCl, and 4) DMEM. h) In vitro accumulative release profiles of PC from PC capsules at pH 7.4 and 5.5. All the values are expressed as mean ± SD, *n* = 3.

Compared to the spherical PC doped calcium carbonate (PC@CaCO_3_) (**Figure** **1**a), the PC capsules were wrinkled and creased under scanning electron microscope (SEM, Figure 1b) and transmission electron microscope (TEM, Figure 1c), with a uniform size of ≈3 µm, similar to PC@CaCO_3_ (Figure 1d). Atomic force microscopy (AFM) imaging (Figure S1, Supporting Information) showed similar folds and collapses observed in Figure 1b, confirming the PC capsules were synthesized successfully. The energy dispersive spectrometer (EDS) was used to analyze elemental changes before and after template removal. As shown in Figure 1e, PC@CaCO_3_ was composed of C, H, O, and Ca elements (left panel), while PC capsules were composed only of C, H, and O elements (right panel), without Ca element. Confocal laser scanning microscopy (CLSM) imaging (Figure 1f) showed the inner hollow structure and good distribution profile of PC capsules in solution. Compared to pure water (Figure S2a, Supporting Information), the PC solution was clear green under CLSM (Figure S2b, Supporting Information), so the appearance of green circles served as proof of capsule formation.

Permeability was analyzed by staining with Fluorescein isothiocyanate‐dextran (FITC‐dextran). CLSM images of the PC capsules showed they were permeable to FITC‐dextran with MW of 40 kDa (Figure S3a, Supporting Information) but impermeable to FITC‐dextran with MW of 500 kDa (Figure S3b, Supporting Information), it is reasonable to speculate that the capsules have semipermeable shells. Stability analysis was performed by mixing the capsules in pure water, phosphate buffered saline (PBS), 0.9% sodium chloride (NaCl), and DMEM for 2 months. In all cases, the capsules maintained their morphology (Figure 1g), similar to that shown in Figure 1b. The release of PC was measured in 10 mM PB (pH 5.5) and 10 mM PBS (pH 7.4). In Figure 1h, PC released from the capsules was very slow, with only 10% released after 3 days, followed by continuous release of less than 75% within 2 months. The capsules serve as containers for storing PC and are capable of slow release, greatly increasing the bioavailability of PC.

### 2.2 ROS Scavenging and RNS Scavenging Ability of PC Capsules

To validate successful synthesis, we investigated the effect of different amounts of PC on the composition of PC capsules. Seven concentrations of PC were doped in CaCO_3_ (5, 10, 20, 30, 40, 60, and 80 mg mL^−1^) (Table S1, Supporting Information). The capsules were formed at concentrations of 10–60 mg mL^−1^ and no capsules were formed at 5 and 80 mg mL^−1^. We hypothesize that calcium chloride complexes with PC form a network when the PC concentration is too high, which interferes with the formation of PC@CaCO_3_. If the PC concentration is too low, it is difficult for the PC to aggregate and form a network, so the capsules cannot be formed. We characterized the capsules formed at the PC concentration of 10, 20, and 30 mg mL^−1^. The inner hollow cores were seen in CLSM images (Figure S4a, Supporting Information), and the folds shown in SEM (Figure S4b, Supporting Information) and TEM images (Figure S4c, Supporting Information) indicated the successful preparation of the capsules. When generating PC@CaCO_3_ templates, the amount of PC doping in CaCO_3_ increased with the initial concentration of PC. Therefore, we characterized the capsules using AFM (Figure S5, Supporting Information). As the amount of PC doping increased, the walls of the capsules became thicker, which was consistent with the observations made by SEM. PC is a class of polyphenols with excellent antioxidant capacity due to the presence of phenolic hydroxyl groups, so we used the Total Antioxidant Capacity kit to investigate the antioxidant properties of capsules at different PC levels. At the concentration of capsules (250 µg mL^−1^), the antioxidant capacity increased slightly with the amount of PC incorporated. When the PC concentration was 60 mg mL^−1^, the capsules showed the highest antioxidant capacity compared to capsules prepared with other concentrations of PC (Figure S6, Supporting Information). Moreover, the capsules prepared at this concentration had good dispersion. Therefore, capsules formed at concentrations of 60 mg mL^−1^ were chosen for subsequent ROS‐scavenging experiments.

**Figure 2:**
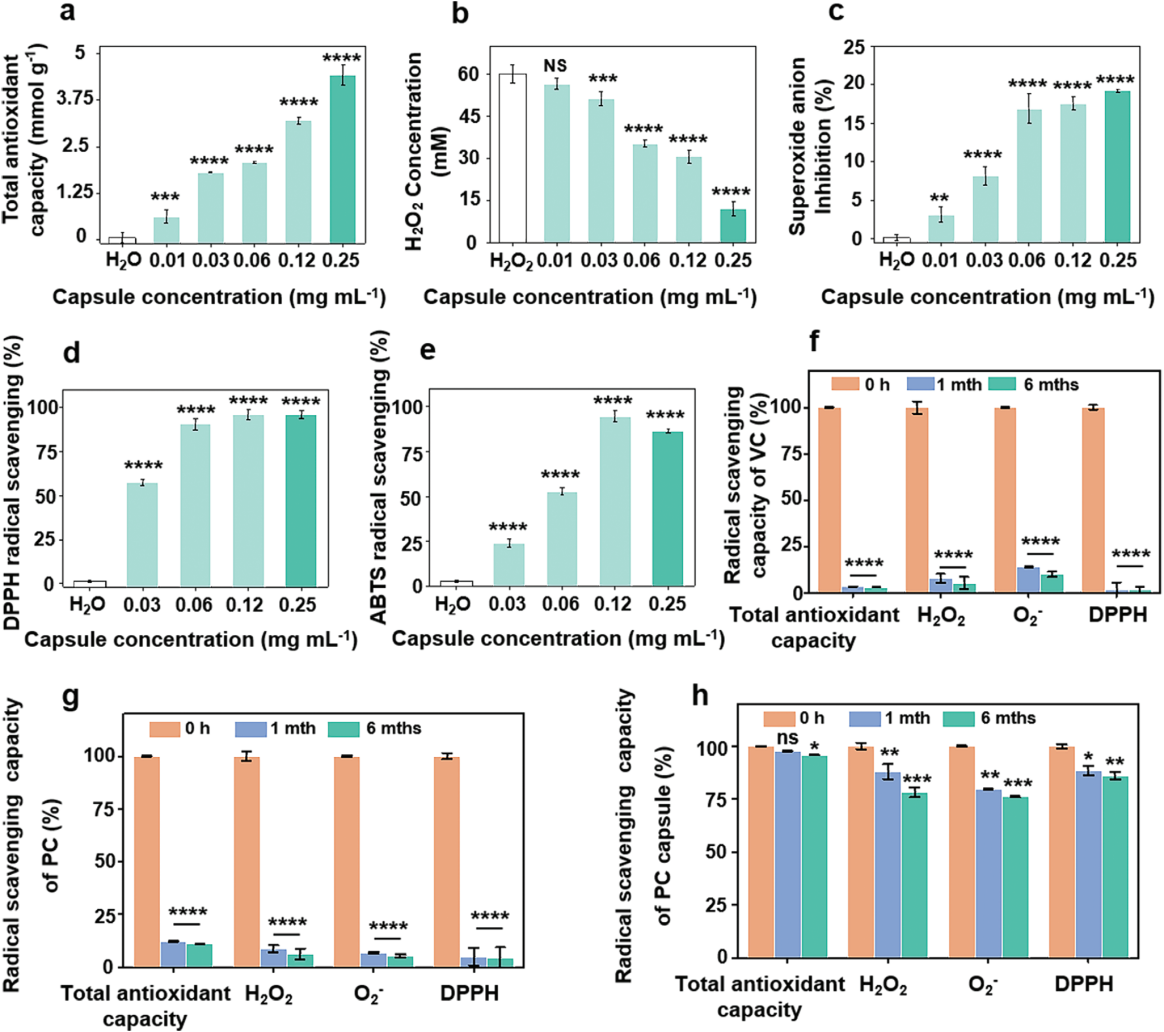
ROS and RNS scavenging ability of PC capsules. a) total antioxidant capacity, b) H_2_O_2_, c) O_2_^−^, d) DPPH, and e) ABTS radical scavenging ability of PC capsules. Storage in an oxidant environment for 0 h, one month, and six months, ROS scavenging capacity of f) VC solution, g) PC solution, and h) PC capsule was measured. ROS scavenging capacity included total antioxidant capacity, H_2_O_2_, O_2_^−^, and DPPH radical scavenging ability. The yellow column represents the effect after 0 h of placement, the blue column represents the effect after one month of placement and the green column represents the effect after six months of placement. *p* values: ns *p* > 0.5, ^**^ *p* < 0.01, ^***^ *p* < 0.001, ^****^ *p* < 0.0001, all the values are expressed as mean ± SD, *n* = 3.

Hydrogen peroxide (H_2_O_2_) and superoxide anion (O_2_^−^) radical assays were selected to investigate the ROS‐scavenging activities of PC capsules. As shown in **Figure** **2**a–c, the capsules exhibited strong ROS‐scavenging activity in a concentration‐dependent manner. More than 4.4 mmol g^−1^ of the free radical (Figure 2a), ≈80% of the total H_2_O_2_ (Figure 2b) and 20% of the O_2_ was decomposed when treated with 250 µg mL^−1^ capsules (Figure 2c). DPPH and ABTS radical assays were used to confirm the RNS scavenging properties of PC capsules. More than 96% and 91% of the DPPH and ABTS free radicals were eliminated by 120 µg mL^−1^ capsules (Figure 2d,e). These results indicated that PC capsules had good ROS and RNS scavenging activities.

Preparation of polyphenols into particles helps protect the phenolic hydroxyl groups from oxidation, thus slowing the loss of antioxidant function and achieving long‐term antioxidant efficacy.^[^ ^19^ ^]^ To demonstrate the long‐term ROS and RNS scavenging capacity of the PC capsules, capsules were placed at room temperature in an oxygenated environment for 0 h, one month and six months. Then total antioxidant capacity assay, H_2_O_2_ scavenging assay, O_2_^−^ scavenging kit, 2‐diphenyl‐2‐picrylhydrazil (DPPH), and 2,2'‐azino‐bis(3‐ethylbenzothiazoline)‐6‐sulphonic acid (ABTS) radical scavenging capacity assay were performed, and the results were shown in Figure S7 (Supporting Information). To compare the antioxidant properties of VC, PC and capsules, the initial scavenging capacity was set to 100% and the ROS and RNS scavenging rates were calculated after placing VC, PC, and capsules for 1 and 6 months. After one month of storage, the total antioxidant ability, H_2_O_2_ scavenging, O_2_^−^ scavenging ability, and DPPH scavenging ability of VC decreased from 100% to 3.44%, 7.79%, 14.16%, and 2.04%, respectively. The total antioxidant ability, H_2_O_2_ scavenging, O_2_^−^ scavenging ability, and DPPH scavenging ability of PC solution decreased from 100% to 12.26%, 8.82%, 7.04%, and 5.1%, respectively. In contrast, the total antioxidant ability, H_2_O_2_ scavenging, O_2_^−^ scavenging ability, and DPPH scavenging ability of capsules were only reduced from 100% to 97.7%, 88%, 79.69%, and 88.57%, respectively (Figure 2f‒h) (the blue column represents the effect after one month of placement). The clearance of ROS from VC and PC solutions after 6 months of placement was comparable to that after 1 month of placement, presumably because clearance had been minimized after 1 month (Figure 2f‒h) (the green column represents the effect after six months of placement). This suggests that PC capsules have better long‐term stable ROS‐clearing ability than PC solution and VC, which may be beneficial in treating chronic inflammatory diseases.

### 2.3 Mechanism of Capsule Formation

**Figure 3:**
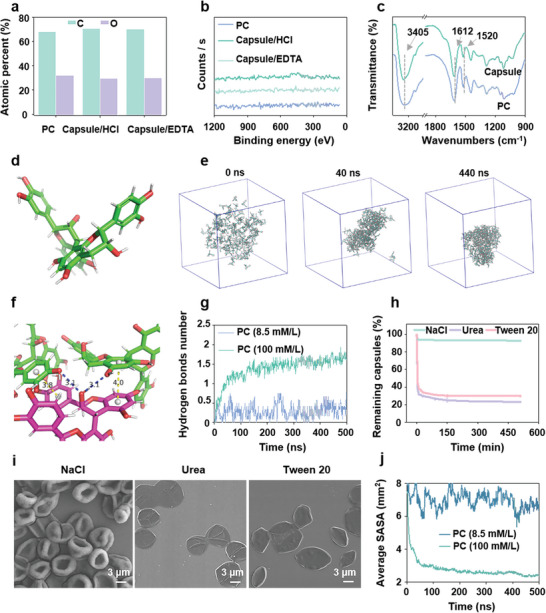
Mechanism of capsule formation. a) XPS data of PC and capsule where capsules were prepared by removing the template by HCl and EDTA, respectively. b) Spectra of Ca^2+^ of PC and capsule where capsules were prepared by removing the template by HCl and EDTA, respectively. c) Fourier transform infrared (FTIR) spectra of PC and capsules. d) The 3D structural formula of PC. e) The representative snapshot (0, 40, and 440 ns) from the all‐atom molecule dynamics simulation trajectory showed the aggregation of high‐density (100 mM L^−1^) PCD molecules. The PCD molecules were shown in licorice. f) The hydrogen bonds (blue dashed line) and π − π interaction (yellow dashed line) among PCD molecules. This structure was obtained from the snapshot of 440 ns in the all‐atom molecule dynamics simulation trajectory showed the aggregation of 100 mM PCD molecules. g) The average hydrogen bond number formed between PCD molecules evolution with time for low‐density PCD (8.5 mM L^−1^, blue line) and high‐density PCD (100 mM L^−1^, red line). h) The percentage of PC capsules remaining after 8 h of incubation with 100 mM of NaCl, urea, or Tween 20, highlighting the dominant interactions of PC. i) SEM images of capsules after 8 h of incubation with 100 mM of NaCl, urea, or Tween 20. j) The solvent accessible area of low‐density (8.5 mM L^−1^) PCD molecules and high‐density (100 mM L^−1^) PCD molecules. All the values are expressed as mean ± SD, *n* = 3.

Inductively coupled plasma‐mass Spectrometry was used to perform the determination of calcium ions in capsules to judge the capsule formation mechanism. (Figure S8a, Supporting Information). The Ca^2+^ content of the capsules was found to be 0%, which was hypothesized that the capsules consisted only of PC. To further verify the absence of Ca^2+^ in the capsules, hydrochloric acid (HCl was replaced with Ethylenediaminetetraacetic acid (EDTA, a strong binder to metal ions) to remove the CaCO_3_ template, and the successful preparation of PC capsules with template removal by EDTA was confirmed by SEM and CLSM (Figure S8b,c, Supporting Information). Then, the formed capsule was further incubated with EDTA overnight to make sure the Ca^2+^ was completely removed, and the capsules were still observed without obvious reduction in amount (Figure S8a, Supporting Information). The X‐ray photonics spectra (XPS) showed that PC solution and capsules formed by EDTA and HCl template removal also consisted of only carbon and oxygen atoms (**Figure** **3**a), and no peak of calcium atoms in XPS confirmed that the capsules did not contain metal ions (Figure 3b). The atomic ratio of carbon and oxygen in PC and capsules were both ≈70% and 30%, respectively, which implied that the capsules had a very similar elemental composition to PC, suggesting that the capsules were constructed from PC only.

Fourier transform infrared (FTIR) spectra was used to determine how PC molecules self‐assemble to form capsules, and the results were shown in Figure 3c. The PC spectrum showed a broad absorption peak at 3305 cm^−1^ ascribed to the phenolic hydroxyl of PC. The absorption band at 1612 cm^−1^ was attributed to the characteristic functional groups of the polyflavonoid moiety, while the absorption band at 1520 cm^−1^ was attributed to the skeletal stretching pattern of the aromatic ring, indicating the prominence of the PC structure.^[^ ^20^ ^]^ FTIR spectra of PC capsules showed similar absorption peaks to PC, except the peak corresponding to hydrogen bonding was shifted to 3400 cm^−1,^ which was ascribed to hydrogen bond formation between the phenolic hydroxyl of PC.^[^ ^21^ ^]^

To further understand the formation mechanism of PC molecules, a series of 500 ns all‐atom molecular dynamics (MD) simulation trajectories were performed to investigate the aggregation behavior of the procyanidin molecules (labeled as PCD in MD simulation). First, the 3D structural formula of the PC molecule was modeled (Figure 3d). Then, the aggregation of PCD molecules under low‐density (8.5 mM L^−1^, unable to form capsule concentration) and high‐density (100 mM L^−1^, could form capsule concentration) conditions were considered. 12 PCD molecules were randomly placed in a box of 13.3^13.3^13.3 nm^3^ to reach the density of 8.5 mM L^−1^. The snapshots obtained from all‐atom MD simulation trajectories showed that the low‐density PCD molecules hardly aggregated in the 200 ns time scale. Some of the PCD molecules aggregated and formed one cluster in the 400 ns. However, this aggregated cluster of PCD molecules was not stable and could not attract other PCD molecules (Figure S9, Supporting Information). In Figure 3e, 128 PCD molecules were randomly placed in a box of 13.3 × 13.3 × 13.3 nm^3^ to reach the density of 100 mM L^−1^. The snapshots obtained from all‐atom MD simulation trajectories show that the majority of high‐density PCD molecules quickly aggregated in the 40 ns time scale. All the PCD molecules aggregated and formed one cluster in the 440 ns. The simulation results indicated the PCD molecules aggregated under certain density conditions. In Figure 3f, the structural detail of aggregated PCD molecules from the 440 ns snapshot from the all‐atom molecule dynamics simulation trajectory showing the aggregation of 100 mM PCD molecules was shown. The PCD molecules aggregated with neighboring PCD molecules via hydrogen bonds and ***π*** − ***π*** interaction. The distance between the oxygen atom of PCD and the oxygen atom of the neighboring PCD molecules was 3.1 Å, indicating stable hydrogen bonds formed between these PCD molecules. Similarly, the distance between the center parallel aromatic ring of neighboring PCD molecules is less than 4 Å, indicating the neighboring PCD molecules aggregated via stable ***π*** − ***π *** interaction. The PCD molecules aggregated with neighboring PCD molecules via 1–2 hydrogen bonds and ***π*** − ***π*** interaction with high structure symmetry. Therefore, we computed the average hydrogen bond number formed with the neighboring molecule per PCD molecule in Figure 3g. For high‐density PCD molecules, the average hydrogen bond number of PCD molecules quickly reached 1 in the 40 ns time scale, indicating that the majority of PCD molecules aggregated, then the average hydrogen bond number of PCD molecules was continuously increased in the 500 ns simulation time scale, which indicated that PCD molecules tightly aggregated and packed in the 500 ns MD simulation trajectory. On the other hand, for the low‐density PCD molecules, the hydrogen bond number of PCD molecules fluctuated ≈0.25, which indicated the PCD molecules were not aggregated despite local contact via hydrogen bonds or ***π*** − ***π*** interaction among the PCD molecules.

Urea, NaCl, and Tween 20 were added to PC capsules to study their decomposition over time, The release of PC in different solvents correlated with capsule degradation, confirmed by UV absorption spectroscopy. Incubation with urea and Tween 20 for 2 h, the PC retention rate in capsules was reduced to 26% and 33%, respectively, due to the disruption of hydrogen bonds and hydrophobic interactions. However, 91% of PC remained in capsules after 8 h in NaCl, suggesting electrostatic interactions were not a primary force in capsule formation (Figure 3h). SEM images of the capsules in different microenvironments were used to further show the degradation of the capsules. The thickness of capsules was significantly reduced after mixing with urea and Tween 20 compared to the initial capsules whereas the thickness of capsules did not change significantly after mixing with NaCl (Figure 3i). Moreover, some capsules maintained their basic morphology, which implied that the π − π interaction was the main force for the maintenance of the capsule structure. The initial morphology of the capsule is formed because of the π − π interactions between the PC molecules, and then the free PC molecules are further bonded by hydrogen bonding and hydrophobic interactions to finally form the PC capsule. Overall, the PC capsules consist of a variety of interacting forces, including hydrogen‐bonding interactions, hydrophobic interactions and π − π interactions with π − π interactions being the main force in the formation of the PC capsule.

Subsequently, we calculated the solvent accessible surface area of the PC molecules, the solvent accessible surface area (SASA) is an indicator to evaluate whether the molecules may have contact with the surrounding solvent. The result is shown in Figure 3j. The vertical coordinate of the graph represents the area of contact between an average PC molecule and the surrounding aqueous solution in nanosquare. It was found that PCs rapidly aggregated at 100 mM concentration and the SASA of a single PC molecule decreased rapidly, whereas PCs remained in the free state with a large SASA at 8.5 mM concentration. Initially, the average SASA of PC for both systems was ≈8 nm^2, and the SASA of PC at 8.5 mM concentration dropped to ≈6 nm^2, while the SASA of PC at another concentration of 100 mM PC could drop to 2 nm^2 at another concentration. The SASA of the non‐aggregated compared to the aggregated state is roughly ≈3 times larger. Therefore, this result also explains why PC molecules are more easily oxidized compared to PC capsules. Moreover, the phenolic hydroxyl groups in PC capsules form hydrogen bonds and π − π interactions, which further protects the phenolic hydroxyl groups from oxidation by oxygen and water in the air and water, so that the antioxidant property of PC capsule is maintained for a longer period, while the antioxidant property of PC molecule is greatly depleted.

In order to verify the universality of this method of capsule preparation, other polyphenols were randomly selected, including gallic acid (GA), catechin (Cat), curcumin, epigallocatechin gallate (EGCG) and tannic acid (TA), to fabricate the capsules by the same strategy. The microscope observed whether capsules were produced, and the morphology of capsules was characterized by SEM microscope. As shown in Figure S10 (Supporting Information), all selected polyphenols could generate capsules by presenting similar morphology to PC capsule, indicating this is a general strategy for producing polyphenol capsules.

### 2.4 ROS‐Responsiveness of PC Capsules In Vitro

**Figure 4:**
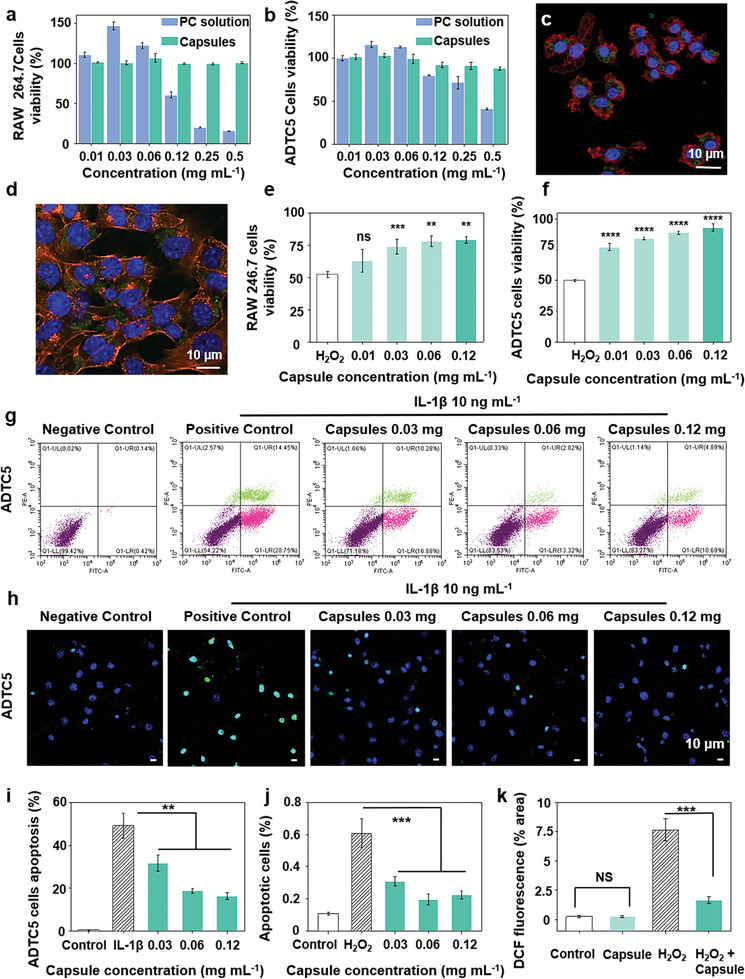
ROS‐responsiveness of PC capsules in cellular. Cell viability of a) RAW 264.7 cells and b) ATDC5 cells after incubating with PC solution and capsules for 24 h. Cellular uptake of capsules in c) RAW 264.7 cells and d) ATDC5 cells. Cell viability induced by H_2_O_2_ of e) RAW 264.7 cells and f) ADTC5 cells. g) Apoptotic chondrocytes were detected using Annexin V‐FITC/PI staining and flow cytometry. h) TUNEL fluorescent images of ADTC5 cells stimulated with IL‐1β and capsules. i) The apoptosis rates of flow cytometry. j) The survival rate of ADTC5 induced by TUNEL fluorescent images. k) Quantification of total fluorescence intensity of DCFH‐DA in RAW 264.7 cells. (P values: ns *p* > 0.5, ^**^ *p* < 0.01, ^***^ *p* < 0.001, ^****^ *p* < 0.0001, all the values are expressed as mean ± SD, *n* = 3).

The right concentration of PC can favor cell growth and proliferation, so the Cell counting kit‐8 (CCK‐8) Fourier transform infrared spectrome assay was used to evaluate the cytotoxicity of PC solution and capsules at different concentrations (0.01–0.5 mg mL^−1^) for 24 and 48 h. As shown in **Figure** **4**a,b, cell viabilities were >85% in the presence of capsules, even at the highest tested concentration (0.5 mg mL^−1^) after 48 h in RAW 264.7 and ADTC5 cells, indicating low cytotoxicity in these cell lines (Figure S11, Supporting Information). Cell viability in the presence of PC solution was <80% at 24 h at the concentration (0.1 mg mL^−1^), indicating that the PC solution is highly toxic. Thus, capsules improve the biocompatibility of PCs, making them amenable to biological applications. RAW 264.7 cells and ADTC5 cells were chosen to assess the ability of capsules to enter cells. As shown in Figure 4c,d, after incubating cells and capsules for 24 h, cells exposed to 120 µg mL^−1^ capsules showed normal polygonal cytoskeleton morphology, indicating good biocompatibility. The green circles localized inside the red cell membrane and outside the blue nucleus showed that the capsules were endocytosed successfully (Figure S12, Supporting Information).

Based on the excellent ROS‐scavenging ability and good biocompatibility demonstrated by PC capsules in vitro, the protective effect of the capsules on cells was monitored by CCK‐8 kit and stimulation with H_2_O_2_ to verify the ROS‐scavenging ability at the cellular level. The results are shown in Figure 4e,f. Compared with the H_2_O_2_ group, the viability of RAW 264.7 cells increased from 52.23% to 79.27%, and ADTC5 increased from 50% to 93.03% after adding the capsules, and the effect was dose‐dependent. The results indicated that PC capsules could remove H_2_O_2_ from cells and attenuate oxidative damage in a variety of cells.

The protective effect of PC capsules on cells was also assessed by flow cytometry to observe the reversion of ADTC5 cells to apoptosis after exposure to IL‐1β. As shown in Figure 4g, compared with the control, the apoptosis rates of ATCD5 cells increased significantly after IL‐1β stimulation, while the addition of PC capsules reduced apoptotic response. Statistical analysis (Figure 4i) revealed that the apoptosis rate in the IL‐1β group reached 49.20%, while the rate in the 0.06 mg mL^−1^ PC capsule group decreased to 18.70%. TUNEL staining showed that compared to the control, green fluorescence was enhanced in the IL‐1β group, and DAPI staining showed that the nuclei were ruptured, indicating that IL‐1β induced apoptosis in ATDC5 cells. Various tested concentrations of PC capsules reduce apoptotic fluorescence and maintain intact nuclei, with only a small number of ruptured nuclei (Figure 4h). Semiquantitative and statistical analysis of fluorescence using ImageJ revealed that the apoptosis rate of the IL‐1β group was 60.80%, while that of the 0.06 mg mL^−1^ PC capsules group decreased to 19.61%. The rate in the 0.12 mg mL^−1^ PC capsules group decreased to 22.67%, revealing a good protective effect in ADTC5 cells (Figure 4j).

To visually evaluate the capsule's ROS‐scavenging ability, a DCFH‐DA fluorescent probe was used to detect ROS in RAW 264.7 cells (Figure S13, Supporting Information). There was no significant difference between the capsule and control groups, indicating that the capsule did not increase oxidative stress. Green fluorescence increased 31.6‐fold with H_2_O_2_ compared to the control, while fluorescence in the H_2_O_2_ + capsule group was significantly reduced by 78.5% (Figure 4k). The addition of H_2_O_2_ increased ROS production, leading to apoptosis. To verify the effect of PC capsules on macrophage inflammation in vitro, expression of interleukin 6 (IL‐6) and tumor necrosis factor‐α (TNF‐α) was evaluated in RAW 264.7 cells using ELISA kits. LPS (2 µg mL^−1^) was added to stimulate an inflammatory response. Cells treated with capsules showed a dose‐dependent reduction in IL‐6 and TNF‐α, with the 0.12 mg mL^−1^ capsule group showing a 1.97‐fold and 1.52‐fold reduction in IL‐6 and TNF‐α compared to the LPS group (Figure S14, Supporting Information). Thus, the PC capsules reduce intracellular ROS and attenuate the cellular inflammatory response, suggesting their therapeutic potential in ROS‐related inflammatory diseases.

### 2.5 In Vivo Therapeutic Efficacy of PC Capsules in Osteoarthritic Mice

**Figure 5:**
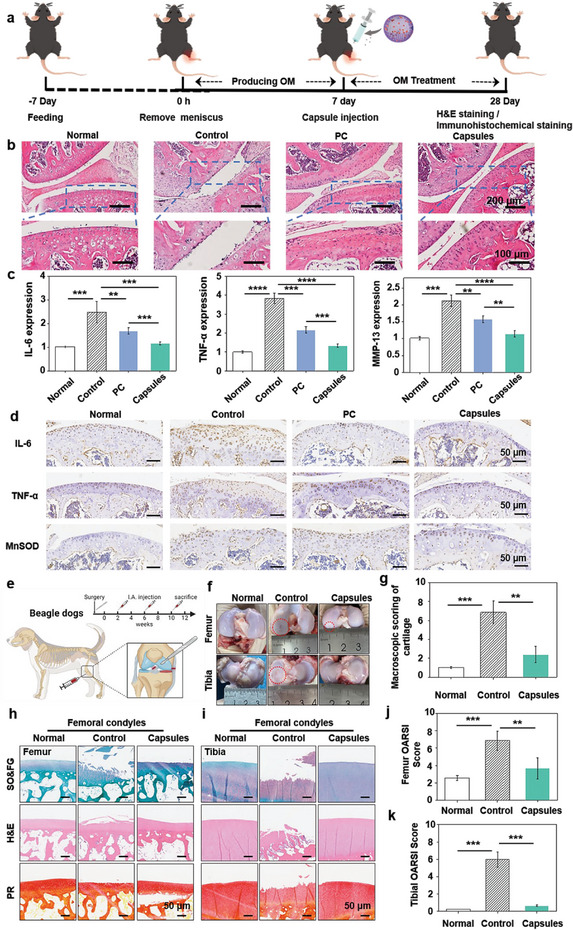
The assessment of therapeutic efficiency in vivo on osteoarthritis. a) Schematic representation of the establishment and treatment of osteoarthritis mice. b) H&E staining of mouse models of osteoarthritis. c) qPCR data in mouse model of osteoarthritis for IL‐6, TNF‐α and MMP‐13. d) Representative immunohistochemical images of PBS, PC and capsule‐treated osteoarthritis sections stained with IL‐6, TNF‐α and MnSOD. e) Schematic representation of the surgical and therapeutic protocols used in a dog model of ACLT‐induced dog OA. f) Macroscopic appearance of the femoral condyle and tibial plateau cartilage and their g) scores after 12 weeks of treatment. (Red dashed line: articular cartilage damage). Representative images of SO&FG, H&E and PR staining in h) dog femoral condyle and i) tibial plateau cartilage after 12 weeks of treatment. Macroscopic cartilage scores based on the OARSI scoring system and for two major weight‐bearing surfaces, including the j) medial femoral plateau and k) medial tibial condyle. (*p* values: ^***^ *p* < 0.001, ^****^ *p* < 0.0001, all the values are expressed as mean ± SD, *n* = 3).

To assess the utility of PC capsules in ROS‐related chronic inflammatory disease, we used a representative disease model of osteoarthritis in mice and beagle dogs.^[^ ^19b^ ^]^ As shown in **Figure** **5**a, the mouse osteoarthritis model was prepared by the Hulth method. To validate the long‐term antioxidant effects of the capsules in vivo, they were injected intra‐articularly once. The hematoxylin‐eosin (H&E) data demonstrated that the OA rats group showed cartilage hypocellularity and erosion, indicating severe damage. The PC solution‐treated group had chondrocytes that were still partially damaged and cellular hyperplasia, while the capsule‐treated group had a smooth, hypercellular cartilage surface with minimal damage (Figure 5b). Then, the bone joints of mice were removed, and only the upper and lower joint heads and joint parts were left as much as possible. RNA was extracted after fragmentation, reverse transcribed into cDNA, and subjected to RT‐PCR Assay. The levels of inflammatory factors in the joints were also assessed, and IL‐6, TNF‐α, and MMP‐13 in the PC group were decreased (38%, 44%, and 23%) versus the control group, but the levels were reduced in the PC capsule‐treated group (59%, 65%, and 45%) (Figure 5c). The results indicated that the PC capsule group could remove the level of ROS from the arthritic site and reduce the level of inflammatory factors compared to the PC solution group. Immunohistochemical staining of IL‐6, TNF‐α, and MnSOD demonstrated the ROS scavenging and anti‐inflammatory effects of PC capsules (Figure 5d). Levels of IL‐6, TNF‐α, and MnSOD in the PBS group were 15.68%, 18.00%, and 17.28%, respectively, versus 12.06%, 10.57%, and 11.94% in the capsule group and 14.38%, 16.03% and 16.07% in the PC group (Figure S15, Supporting Information). Subsequently, the secretion of collagen II and aggrecan in the PC capsule group was determined by immunofluorescence staining. As shown in Figure S16 (Supporting Information), the relative contents of collagen II and aggrecan in the PC capsule group were closest to those in the normal group, both exceeding 60%. In the PC solution group, the relative content of collagen II and aggrecan was more than 50%. In addition, there was a significant difference in the levels of collagen II and aggrecan between the PC capsule group and the PC solution group, indicating that the PC capsule group had the better chondrocyte‐promoting activity.

Based on the in vivo therapeutic efficacy of PC capsules in mice, the therapeutic potential of the PC capsule was further evaluated in enough models using a preclinical large animal model, where the cartilage characteristics of dogs were same to those of humans. Male beagles underwent ACLT followed by single joint treatment with PBS and PC capsules (Figure 5e). According to the dog OA scoring system published by the OARSI Histopathology Initiative, cartilage damage was evident in the untreated PC capsule groups after 12 weeks of treatment. The most severe osteoarticular inflammation occurred mainly in the central area of the anterior femoral condyle and the weight‐bearing area of the tibial plateau (Figure 5f,g). In contrast, similar to the findings in mice, a smooth surface was observed in the PC capsule group.

Pathological sections were also used to observe the softness of the diseased dog knee joint (Figure 5h,i). In the negative control group, a smooth cartilage surface with all areas intact was observed by SO&FG and H&E staining. In the OA model, cartilage degeneration occurs over a wide area, with the femoral condyle and the tibial plateau showing different patterns of cartilage damage. Among them, the femoral condyle had more proteoglycan loss and surface fibrillation, while the tibial plateau had less proteoglycan loss but more surface structure damage. However, after 12 weeks of treatment, the knees treated with the PC capsules showed mild signs of degeneration and preserved cartilage integrity. In addition, PR staining showed that the collagen structure of the knee of the dog with OA was disturbed, particularly in the superficial layer of the cartilage. In contrast, PR staining in the PC capsule group showed the expected normal distribution. It was very similar to that in the sham group.

The microscopic grading of the synovial changes in the dogs was also evaluated using the OARSI scoring system and the Mankin scoring system in accordance with the dog scoring system (Figure 5j,k). We observed an increase in the number of synovial cell layers and even villous hyperplasia in the joints of OA dogs treated with PC capsules in comparison to the negative control group. Taken together, these data suggest that PC capsules act as a long‐term agent that directly reduces cartilage degradation and provides multiple benefits to affected joints in preclinical dog models of OA. We have confirmed the potential utility of PC capsules in treating chronic inflammatory diseases.

### 2.6 PC Capsules Reduce ROS via the MAPK Pathway to Exert a Long‐Term Anti‐Inflammatory Effect

**Figure 6:**
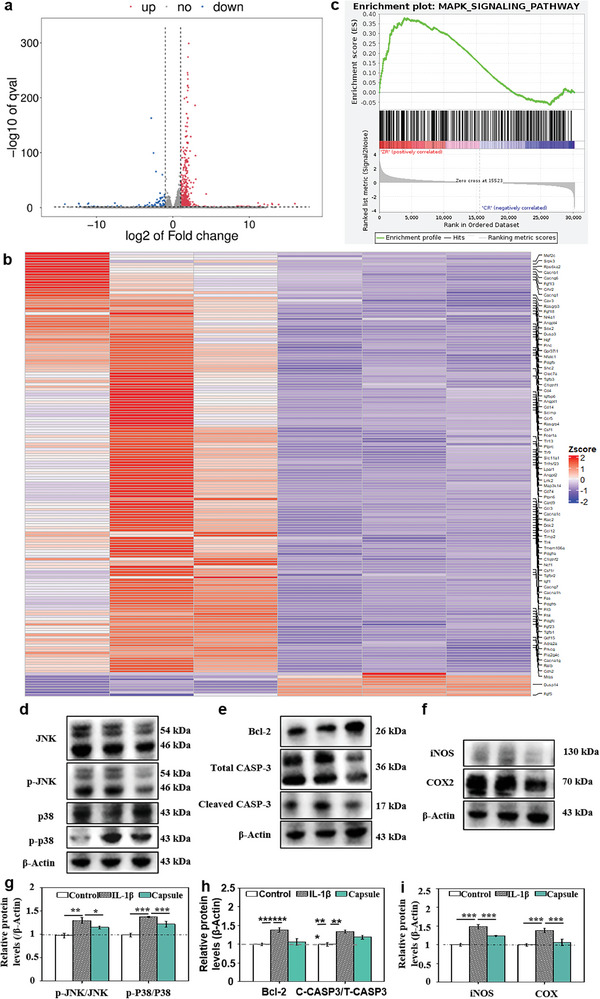
PC capsules inhibited MAPK expression in osteoarthritis. a) Volcano plot displaying DEGs between capsule‐ and blank control‐treated osteoarthritic mice. b) Heatmap of DEGs in the osteoarthritis mouse model. c) GSEA analysis of the MAPK gene set in the osteoarthritic mouse model. ADTC5 cells were exposed to IL‐1β then treated with PC capsules. d) Phosphorylation of proteins involved in MAPK signaling (JNK, p38) as determined by western blotting. e) Expression of p38, p‐P38, total caspase3, cleaved caspase3, and Bcl‐2. f) p38, p‐P38, iNOS, and COX‐2 was assessed by western blotting. g) Quantification of (d). h) Quantitation of (e). i) western blotting band quantitation of (f). (*p* values: ^**^ *p* < 0.01, ^***^ *p* < 0.001, all values are expressed as mean ± SD, *n* = 3).

Transcriptome analysis via high‐quality reads mapping to the Mus musculus reference genome revealed consistent mapping rates of >97% for osteoarthritis samples, providing a comprehensive characterization of PC capsule effects versus untreated mouse models. The transcriptome data obtained from the two models were of high quality as assessed by Pearson's correlation test (Figure S17, Supporting Information), fragments per kilobase of transcript per million fragments mapped (FPKM) analysis (Figure S18, Supporting Information), and gene expression density analysis (Figure S19, Supporting Information). We identified 89 878 transcripts in the osteoarthritis models, corresponding to 30 250 genes. We identified 591 (488 upregulated and 103 downregulated, **Figure** **6**a) significantly differentially expressed genes in the osteoarthritis models.

Elevated intracellular ROS cause damage to lipids, proteins, and DNA and are linked to a myriad of pathologies. The imbalance between ROS production and the mitigation of oxidative stress can lead to chronic inflammation.^[^ ^22^ ^]^ Hydrogen peroxide is required for many cellular pathways regulated by MAPK, NF‐κB, and PI3K signaling.^[^ ^23^ ^]^ We categorized the differentially expressed genes (DEGs) by GO annotation (Figure S20, Supporting Information), then performed cluster analysis of ROS‐related DEGs in the chronic inflammation models and observed significant aggregation of the MAPK pathway. Notably, most MAPK signaling genes were inhibited by PC capsules (Figure 6b). GSEA analysis was used to analyze DEGs based on the KEGG gene sets from MSigDB v7.4 and showed that MAPK_SIGNALING_PATHWAY was significantly downregulated by PC capsules. For the osteoarthritis model, GSEA tagged 56 core enrichment genes, seven of which were DEGs (Figure 6c). MAPK signaling is the principal pathway modulating inflammatory responses and directly regulating ROS metabolism in chronic inflammation.^[^ ^24^ ^]^ MAPK inhibitors have been well studied in the treatment of various chronic inflammatory diseases and have more than 20 candidates in clinical trials.^[^ ^25^ ^]^ Previous studies have demonstrated the favorable effect of MAPK inhibitors alone or combined with metformin in diabetes.^[^ ^26^ ^]^ PC capsules demonstrated effective therapy with one administration, low cytotoxicity, and potential for MAPK inhibitor development, offering an alternative approach to the frequent and lengthy treatments required for osteoarthritis and other chronic inflammatory conditions.^[^ ^25^, ^27^ ^]^

Transcriptome analysis showed that PC capsules regulate MAPK in OA models. p38 and JNK are MAPK‐mediated signaling cascades that regulate the inflammatory response and apoptosis.^[^ ^28^ ^]^ We aimed to determine whether PC capsules affect the MAPK‐p38 or MAPK‐JNK signaling pathways. Phosphorylation of p38 and JNK was dramatically upregulated, and PC capsules restored p38 and JNK phosphorylation in IL‐1β‐inducing inflammatory cell models (Figure 6d,g). Immunohistochemistry results were consistent with western blotting (Figure S21, Supporting Information). MPAK‐p38 signaling promotes apoptosis in the inflammatory response.^[^ ^29^ ^]^ The flow cytometry results suggest that PC capsules suppress apoptotic IL‐1β‐induced inflammatory response (Figure 4g). We measured changes in apoptosis‐associated proteins Bcl‐2 and cleaved caspase 3, which were induced by IL‐1β and restored to normal levels by PC capsules (Figure 6f,i). MPAK‐p38 directly regulates the transcription of the inflammatory markers iNOS and COX‐2 and our results showed the same trends in p‐P38, iNOS, and COX‐2 (Figure 6d,f).^[^ ^30^ ^]^ These results suggest that PC capsules inhibit apoptosis via MAPK‐p38 but not MAPK‐JNK to exert its anti‐inflammatory effects.

### 2.7 Biocompatibility Evaluation of PC and Capsules

We evaluated the effect of PC capsules (10 mg kg^−1^, 5‐fold higher concentration than that used for the treatment of osteoarthritis) on the histopathology of major organs in healthy mice to characterize the biocompatibility in vivo. As shown in Figure S22 (Supporting Information), no necrosis, congestion, or hemorrhage was observed in the heart, liver, spleen, and lung at 28 days after injection of PC capsules in the joint cavity. All results confirmed that the PC capsules have negligible short‐term and long‐term in vivo toxicity.

The biocompatibility of PC capsules was also evaluated by intraperitoneal injection of mice with various concentrations of PC solution and capsules. Serum levels of liver and kidney function markers were assessed at 24 h, 72 h, and 7 days. As demonstrated in Figure S23a–d (Supporting Information), the levels of AST, ALT, blood urea nitrogen, and CRE at all concentrations of PC and capsules were similar to the control group, indicating that the PC capsules had no significant biotoxicity to the liver or kidney. Blood analysis 7 days after injection with PC solution and capsules (Figure S23e–i, Supporting Information) showed no significant differences compared to the control. All results confirm that the synthetic PC capsules exhibit negligible toxicity in vivo.

## 3 Discussion

**Figure 7:**
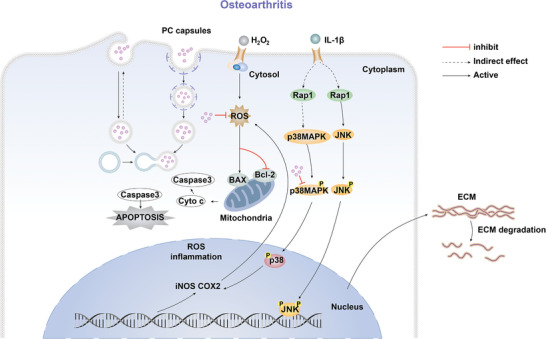
Schematic illustration of the potential mechanism by which intracellular PC capsules exert anti‐inflammatory effects by inhibiting apoptosis. Capsules could regulate the MAPK‐p38 signaling pathway in the Osteoarthritis of mice.

Our results have conclusively demonstrated that MAPK signaling is regulated by PC capsules in mouse models of osteoarthritis (**Figure** **7**). Rat sarcoma (Ras) and Ras‐related protein 1 (Rap1) signaling regulate cartilage degeneration closely related to osteoarthritis disease progression.^[^ ^31^ ^]^ Recent studies have shown that neutrophils enter the lesion early in osteoarthritic inflammation, and the resulting neutrophil extracellular traps are closely related to osteoarthritis.^[^ ^32^ ^]^ Our results are consistent with reports that suggest that in addition to MAPK signaling, Ras and neutrophil signaling are also affected by PC capsules in osteoarthritis (Figure S24, Supporting Information). PC capsules coregulate MAPK signaling during inflammation and influence unique signaling pathways in different disease models depending on the disease type.

PC is a class of polyphenolic compounds widely distributed in plants and with strong antioxidant properties, scavenging excess free radicals and protecting lipids from peroxide damage. As such, they have great potential for use in food and supplements as well as pharmaceuticals and cosmetics.^[^ ^33^ ^]^ Anti‐inflammatory studies of PC like the one here have been performed in osteoarthritis diseases. Min et al. reported that PC significantly attenuates MIA (monosodium iodoacetate)‐induced osteoarthritic injury in rats by inhibiting cartilage damage and osteoclastogenesis.^[^ ^34^ ^]^ However, the therapeutic effects observed in these studies were only achieved by very high doses and multiple injections (such as 100 or 300 mg kg^−1^ PC in osteoarthritis), though they did not explore whether these high doses would lead to toxic side effects.^[^ ^34^, ^35^ ^]^ In this study, the novel PC capsules achieved the same anti‐inflammatory efficacy in osteoarthritis after only one injection and at a much lower dose, greatly improving their clinical potential. The reason for the high efficiency in treating osteoarthritis might be the increased bioavailability and slow metabolism of PC capsules.

To gain a comprehensive understanding of how PC capsules repair osteoarthritis after a single injection, it is important to identify its site of action within the cell.^[^ ^36^ ^]^ Subcellular localization indicated that PC capsules localized to the cytoplasm (Figure 3d). In addition, the PC capsules were trapped in lysosomes at the beginning of endocytosis, and likely did not escape even after endocytosis 7 days later, indicating that the majority of intracellular PC capsules were in lysosomes (Figure S25, Supporting Information). Unlike other reported metal‐polyphenol materials, which are released quickly at decreased pH, the PC capsules slowed release with decreasing pH, suggesting that endocytosis may achieve long‐lasting, slow release in the lysosome.^[^ ^32^, ^37^ ^]^ The slow‐release profile of PC capsules in the lysosome microenvironment (pH = 4.6) suggests the PC can sustain an intracellular concentration for a long period. We understood the concentration of intracellular PC is very low, but was sufficient to modulate the inflammatory response by directly activating or inhibiting multiple intracellular inflammatory signals.^[^ ^38^ ^]^ Other data (Figure 3a,b) demonstrated that the cell viability and proliferation of RAW 264.7 and ADTC5 cells were enhanced even at a very low PC concentration (<30 µg mL^−1^). Under physiological conditions, the PC capsules also displayed sustained release (Figure 1h), providing extracellular PC at low concentrations to modulate the cellular response through specific channels in the cellular membrane to achieve anti‐inflammatory effects.^[^ ^39^ ^]^ In addition, the persistence of long‐acting antioxidant capacity offered another avenue by which PC capsules modulated the microenvironment of chronic disease in osteoarthritis. This study focused on the use of PC capsules in chronic diseases predominantly limited to localized tissue sites. PC capsules were applied in osteoarthritis, where they were localized to the damaged osteoarticular cavities. The PC capsules provided extraordinary performance in curing osteoarthritis.

The described method for producing PC capsules is straightforward and efficient, yielding highly pure PC capsules with robust antioxidant properties for sustained release. The matrix‐free capsules avoid the use of toxic materials and maintain the medical benefits of PC. The high PC content in the capsules ensures prolonged activity and therapeutic efficacy. In osteoarthritis diseases, PC capsules selectively target affected tissue and exhibit good biocompatibility and stability, suggesting broad application potential in treating chronic inflammatory conditions involving the circulatory system.^[^ ^40^ ^]^ Future studies should include more circulatory‐related inflammatory models to enhance the clinical prospects of PC capsules.

## 4 Conclusion

We demonstrate a simple and versatile strategy to generate stable and well‐dispersed polyphenol capsules that achieve ultra‐slow release (2 months) and long‐lasting free radical scavenging (6 months) of proanthocyanidins. These capsules are biocompatible, efficiently absorbed by cells, and modulate the MAPK signaling pathway to protect cells from inflammatory damage and accelerate osteoarthritis healing. The excellent efficacy of the capsules can be demonstrated in a single dose, which is a significant advantage over the long‐term use of traditional drugs. This strategy is expected to facilitate the large‐scale production and clinical application of polyphenol capsules for the treatment of other chronic inflammatory diseases

## 5 Experimental Section

### Reagents

Calcium chloride anhydrous (CaCl_2_, 96.0%), sodium carbonate anhydrous (Na_2_CO_3_, ≥ 99.8%), procyanidins (PC), hydrochloric acid (HCl), hydrogen peroxide solution (H_2_O_2_) and xylenol orange indicator were purchased from Aladdin Industrial Corporation (Shanghai, China). The total antioxidant capacity assay kit, superoxide assay kit, TUNEL apoptosis assay kit, Annexin V‐FITC/PI apoptosis kit, and BCA protein concentration test kit were purchased from Beyotime Biotechnology (Shanghai, China). CCK‐8 reagents were purchased from Dojindo Molecular Technologies (Shanghai, China). DPPH radical scavenging capacity assay kit, ABTS radical scavenging capacity assay kit, 2,7‐dichlorodihydrofluorescein diacetate (DCFH‐DA), 4′,6‐diamidino‐2‐phenylindole dihydrochloride (DAPI, ≥ 90%) were obtained from Beijing Solarbio Science & Technology Co., Ltd. Recombinant Murine IL‐1β, Lipopolysaccharide (LPS), and Streptozotocin were purchased from Sigma–Aldrich (Shanghai, China). Anti‐mouse ELISA kits for IL‐6 and TNF‐α were obtained from Multisciences. Monoclonal antibodies for Bcl‐2, BAX, and Caspase 3/p17/p19 were obtained from Thermo Fisher Scientific (Beijing, China). Dulbecco antimurine antibodies p38, p‐p38, JNK, P‐JNK, Aggrecan, Collagen II, INOS, COX2, MnSOD, and B‐Actin Monoclonal antibody were purchased from Proteintech (Wuhan, China). Serum‐free Cell cryopreservation solution (C40050) and universal antibody diluent (WB100D) were purchased from New Cell & Molecular Biotech. Cell culture flask (TCF011025), cell culture dish (TCP010006, TCP010024 and TCP010096) and PCR tubes (PCR520200) were purchased from Guangzhou Jet Bio‐Filtration Co., Ltd. Glass‐bottom cell culture plates (801 002 and 801 004) and frozen storage tube (612 521) were purchased from NEST Biotechnology Co. Ltd. (Wuxi, China). All reagents and materials were used without further purification.

### Characterization

Scanning electron microscope (SEM, SU8010 HITACHI) and Transmission electron microscope (TEM, FEI Talos F200S microscope) were used to observe the morphology of microcapsules. Confocal Laser Scanning Microscopy (CLSM) mounted on a Nikon A1 apparatus was used to investigate the micrographs of capsules and cells. Optical microscope images were taken with a NIKON Ni‐U microscope and 10 × lenses bright field were used. The surface roughness and thickness of capsules were determined via Bruker Dimension Icon Atomic Force Microscopy (AFM). Fourier transform infrared spectrometer (Bruker TENSOR II) (FTIR) was used to measure the infrared spectra of capsules and the materials for preparing capsules. X‐ray photonics spectra (XPS) were obtained on a Thermo‐Electron ESCALAB 250 spectrometer. Inductively coupled plasma‐mass Spectrometry (ICP‐MS) was used to test the presence of calcium in capsules. UV−vis spectra were determined through an UV−vis‐near red spectrometer (CARY 5000, USA). The sample absorbance was detected by the Lambda 25 spectrophotometer from PerkinElmer (USA). Enzyme‐linked immunoassay (Tecan), flow cytometry (Becton‐Dickinson), Vortex mixer Bioland (China), Roche light cycler 480 fluorescence quantitative PCR instrument (Roche), gel imaging system, and protein electrophoresis instrument (BIO‐RAD) were used to detect proteins and DNA in cells.

### Synthesis of PC Capsules

Briefly, 800 mg PC was dissolved in deionized water (10 mL) and 2 M NaOH was added to adjust pH to 9 to dissolve PC. The preparation method of PC@CaCO_3_ and capsules was as follows. First, the CaCl_2_ solution (3 mL of 0.33 M) was poured into a conical flask where there had Na_2_CO_3_ (2 mL of 0.5 M) and PC (1 mL) with set concentrations (SCon, 5, 10, 20, 40, 40, 60, and 80 mg mL^−1^). After stirring for 40 s at 1200 rpm and aging for another 15 min without any disturbance, the formed microparticles were collected at 1000 rpm, 3 min and washed with deionized water, the centrifugation and washing process was repeated three times. After removing the supernatant, the PC capsules were prepared by adding 50 mM EDTA and vortexing for 4 h or adding 1 M HCl and vortexing for 10 s. Finally, PC capsules were obtained via 3000 rpm, 5 min, and washed with deionized water. The prepared PC capsules were used for further experiments.

### Molecular Dynamics Simulation

The procyanidin molecules (labeled as PCD in MD simulation) were solvated in a water box of 13.3 × 13.3 × 13.3 nm^3^. The CHARMM‐GUI server was used to generate the configuration, topology, and parameter files with the CHARMM36 m force field. For system I and system II, 8.5 and 100 mM of PCD molecules were added for MD simulation, respectively. In addition to the PCD molecules, the simulation system I and system II included ≈69 782 water molecules, 195 sodium, and 195 chloride ions (mimicking the 150 mM NaCl present in the PCD molecules buffer), totaling ≈210 000 atoms for each system. The cubic periodic boundary condition was used during the simulations and the van der Waals interaction was switched off from 1 to 1.2 nm. The Particle Mesh Ewald (PME) method calculated the long‐range electrostatic interactions. Energy minimization was done using the steepest descent algorithm, followed by a 0.4 ns NVT (constant particle number, volume, and temperature) and a 20 ns NPT (constant particle number, pressure, and temperature) equilibration simulation by gradually decreasing force restraints from 1000 kJ mol^−1^ nm^−2^ to 400 mol^−1^ nm^−2^ (for NVT stage) and 400 kJ mol^−1^ nm^−2^ to 40 mol^−1^ nm^−2^ (for NPT stage). All force restraints were removed after the equilibration steps, and the MD simulation was performed in the NPT ensemble. The 500 ns all‐atom MD simulation trajectory was performed for the simulation system I and system II by using GROMACS 2021 at 300 K using a time step 2 fs.

### Release Performance of PC Capsules

The release of PC capsules was assessed using UV–vis spectroscopy. PC capsules (1 mg) were incubated in 10 mM PB buffer (pH 5.5) and 10 mM PBS buffer (pH 7.4), with samples collected at various time points. After centrifugation, the supernatants were analyzed for UV absorption. Fresh buffer was added before resuming incubation. The release rate was calculated as the cumulative release at each time point, expressed as a percentage. This process was repeated for control and sample groups, with three replicate experiments performed.

### Free Radical Scavenging Assays

Total Antioxidant Capacity Assay Kit with FRAP method, H_2_O_2_ scavenging kit, DPPH radical scavenging Capacity Assay Kit and ABTS radical scavenging Capacity Assay Kit were used to detect the ROS scavenging capacity. The experiment was followed by the manufactured.

### Cells and Animals

The RAW 264.7 cell line and ADTC5 cell line were obtained from the Culture Collection of the Chinese Academy of Sciences (Shanghai, China). DMEM medium (PM150210), penicillin‐streptomycin solution (PB180120) was purchased from Pricella Life Science & Technology Co., Ltd. Other materials for cell culture were purchased from Thermo Fisher Scientific and Sigma–Aldrich (Shanghai, China). The cells were cultured at 37 °C in a CO_2_ incubator. Male C57BL/6 mice and male beagle dogs were purchased from Shanghai SLAC Laboratory Animal Co., Ltd (SLAC). All animal experiments in this study were approved by the Wenzhou Medical University Institutional Animal Care and Use Committee (Approval number: SYXK 2015‐009).

### Cytotoxicity Assay of PC Capsules In Vitro

The cytotoxicity of the PC solution and PC capsules was evaluated by cell counting kit‐8 (CCK‐8) assay. RAW 264.7 and ADTC5 cells were cultured in 96‐well culture plates (5 × 10^3^ cells per well). After 24 h incubation, PC solution or PC capsules with varying concentrations (0.01, 0.03, 0.06, 0.12, 0.25, and 0.5 mg mL^−1^) were added into the cells for another 24 and 48 h. Then, cells were incubated for 2 h at 37 °C with 10 µL of CCK‐8. Finally, the absorbance at 450 nm was tested via a spectrophotometer to determine cell viability.

### Internalization of PC Capsules

Briefly, At the selected time point (24 h), RAW 264.7 cells and ADTC5 cells were cultured in 24‐well culture plates (5 × 10^4^ cells per well), After 24 h incubation, PC capsules with different concentrations were added. Culturing for another 24 h, the cells were washed with PBS and fixated with 4% paraformaldehyde for 10 min. Then the cells were washed with PBS and stained with DAPI for another 10 min. After permeabilization of the membrane with 0.5% Triton X‐100 in PBS, cells were stained with Rhodamine Phalloidin. All cell Samples were observed with CLSM.

### Intracellular ROS Scavenging Activity

RAW 264.7 and ADTC5 cells were seeded in 96‐well culture plates (5 × 10^3^ cells per well). After 24 h incubation, PC capsules were added with different concentrations. Culturing for 12 h for RAW 264.7 cells and 24 h for ADTC5 cells, the medium was removed and 0.5 mM H_2_O_2_ was added to the cells and then incubated for another 30 min. The cells were washed with PBS and replaced with fresh medium. After 24 h incubation, cell viability was detected by the CCK‐8 assay described previously. 500 µM H_2_O_2_ treated cells were used as a control.

### Cell Apoptosis was Detected by Flow Cytometry and TUNEL Kit

ADTC5 cells were seeded in 24‐well culture plates (5 × 10^3^ cells per well). After 24 h incubation, ADTC5 cells were treated with 10 ng mL^−1^ of IL‐1β for 24 h. Then, PC capsules were added with different concentrations for another 12 h incubation. According to the reagent supplier's instructions, cells from different treatment groups were collected and washed twice using PBS, add 5 µL Annexin V‐FITC and 10 µL PI per tube, mixed gently, and incubated for 5 min at room temperature. Then flow cytometry was used and the results were analyzed by FlowJo software. For the TUNEL kit, the adherent cells were washed once with PBS and fixed with 4% paraformaldehyde for 30 min. The TUNEL test solution was prepared according to the reagent vendor's instructions. After adding PBS containing 0.3% Triton X‐100, 50 µL TUNEL test solution was added to the sample and incubated for 60 min at 37 °C protected from light. The results were performed under the fluorescence microscope.

### Western Blot Analysis

The protein was extracted via RIPA buffer and the concentrations were determined with a BCA kit. Then the protein was separated via 10% or 12% SDS‐PAGE and transferred to 0.22 µm PVDF membrane. The protein was detected via one‐step or two‐step antibody immunoblotting color development. β‐actin was used as internal reference protein to normalize total protein expression and phosphorylated proteins were calculated as a ratio of phosphorylated‐to‐total protein.

### Establishment of Mouse Model of OA

24 mice were randomly divided into four groups (six rats per group) as follows: sham group (20 µL PBS solution); OA group (medial meniscectomy, and 20 µL PBS solution); PC solution group (medial meniscectomy and 20 µL PC solution (10 mg kg^−1^)); and PC capsules group (medial meniscectomy and 20 µL PC capsules (10 mg kg^−1^)). All groups were given drugs once after surgery. Six weeks age male mice (C57BL/6, weighted ≈20 g) were anesthetized with 0.3% sodium pentobarbital for abdominal anesthesia. The OA model was established by removing the medial meniscus of the knees. The medial meniscus was resected rapidly, and the wound was closed by absorbable sutures. The mice in the sham group also underwent incision and suturing without meniscectomy. The mice were euthanized after surgery 4 weeks and knees were collected for histological analyses and immunohistochemistry staining.

### Dog Model of OA

Nine healthy male beagle dogs (body weight 16.83 ± 1.49 kg) were randomly assigned to three groups (negative control group, OA group, and capsule treatment group, *n* = 3). Knee surgery was performed on the hind limbs of the dogs. Animals were acclimatized in the rearing facility for 7 days prior to manipulation. After a 12‐h fast, the dogs were sedate with atropine (0.02 mg kg^−1^, subcutaneous) before induction. Anesthesia was induced by a mixture of ketamine (10.0 mg kg^−1^, intramuscular injection (im)) and xylazine (1.0 mg kg^−1^, im), followed by tracheal intubation. General anesthesia was maintained by inhalation of 1.5% isoflurane. The right knee joint was shaved and prepared with povidone iodine for sterile surgery. A medial parapatellar incision was then made, the medial collateral and anterior cruciate ligaments were excised, and knee instability was confirmed after the anterior drawer test. The joint was rinsed thoroughly, the incision was sutured layer by layer, and then bandaged. All animals received prophylactic antibiotics (cefazolin, 40 mg kg^−1^, i.v.) and were allowed to roam the outdoor range for 2 h per day. Intra‐articular injections were given every 4 weeks from 14 days after surgery. Each joint was injected with 100 μ (1 mg per milliliter,0.1 mL) of PC capsules. After 12 weeks of treatment, animals were euthanized under general anesthesia. The knee tissues were collected, fixed, decalcified, embedded, and sectioned. H&E staining, Safranin O‐fast green staining, and Sirius red staining were performed for pathological analysis.

### Histopathology Analysis

The collected connective tissues and skin tissues were fixed with 4% paraformaldehyde. The collected connective tissues were decalcified by 10% EDTA for 4 weeks at 4 °C before embedding. Then the tissues were embedded in paraffin, and sectioned into 8 µm. Histological and immunohistochemistry staining was performed to assess morphological changes and inflamed parts.

### Transcriptome Analysis

Total RNA from osteoarthritis mouse models was extracted using Trizol reagent and mRNA was purified from total RNA using Dynabeads Oligo (dT) (Thermo Fisher, CA, USA) with two rounds of purification. The library was constructed and sequenced on an Illumina Novaseq 6000 sequencer (LC‐Bio Technology CO., Ltd., Hangzhou, China) using the paired‐end RNA‐seq approach. Clean reads were filtered by Cutadapt and aligned to the Mus musculus reference genome. StringTie and ballgown were used to estimate the expression levels of all transcripts and perform expression abundance for mRNAs by calculating the FPKM (fragment per kilobase of transcript per million mapped reads) value. Genes differential expression analysis was performed by DESeq2 software between two different groups. The genes with the parameter of false discovery rate (FDR) below 0.05 and absolute fold change ≥2 were considered differentially expressed genes. Differentially expressed genes were then subjected to enrichment analysis of GO functions and KEGG pathways.

### Statistical Analysis

All quantitative data were expressed as the mean ± standard error (SD). The statistical differences between groups were determined using one‐way ANOVA followed by Tukey's test analysis (GraphPad Prism 8.0). A statistically significant difference was considered at a minimal level of significance of p < 0.05, and denoted as ns p > 0.05, * *p* < 0.05, ** *p* < 0.01, *** *p* < 0.001, **** *p* < 0.0001.

## Conflict of Interest

The authors declare no conflict of interest.

## Author Contributions

S.W. and Z.S. contributed equally to this work. S.W., L.S. and X.Z. performed conceptualization. Z.S., D.Y., S.W., and L.S. performed methodology. L.L., Y.W., Y.G. and C.Z. performed formal analysis. S.W. and L.S. wrote the original draft. All authors wrote, reviewed and edited. C.Z., L.L. and X.Z. performed funding acquisition. L.L., Y.G., C.Z., and X.Z. provide resources.

## Supporting information



Supporting Information

## Data Availability

The data that support the findings of this study are available on request from the corresponding author. The data are not publicly available due to privacy or ethical restrictions.
