# Supplementary material for: Ultra‐Long‐Term Anti‐Inflammatory Polyphenol Capsule to Remodel the Microenvironment for Accelerating Osteoarthritis Healing by Single Dosage
Source: Adv Sci (Weinh). 2024 Nov 18;11(48):2407425. doi: 10.1002/advs.202407425 (PMC11672291; doi:10.1002/advs.202407425)
Supplement: Supplementary file 1 — Supporting Information [file ADVS-11-2407425-s001.docx]

Supporting Information

Ultra-long-term Anti-inflammatory Polyphenol Capsule to Remodel the Microenvironment for Accelerating Osteoarthritis Healing by Single Dosage

*Shaoyin Wei ^1, 2, †^, Zeyu Shou ^3, †^, Dong Yang ^1, 2^, Linxiao Sun ^4^, Yan Guo ^5^, Yang Wang ^2^, Xingjie Zan ^2 *^, Lianxin Li ^6 *^, Chunwu Zhang ^3 *^*

S. Wei, D. Yang,

School of Ophthalmology and Optometry, Eye Hospital, School of Biomedical Engineering, Wenzhou Medical University, Wenzhou, 325035, China

S. Wei, D. Yang, Y. Wang, X. Zan

Wenzhou Key Laboratory of Perioperative Medicine, Wenzhou Institute, University of Chinese Academy of Sciences, 325001, China

E-mail: zanxj@ ucas.ac.cn (X. Zan);

Z. Shou, C. Zhang

The First Affiliated Hospital of Wenzhou Medical University, Wenzhou 325000, China

E-mail: zcw6681@wmu.edu.cn (C. Zhang).

L. Sun

Key Laboratory of Diagnosis and Treatment of Severe Hepato-Pancreatic Diseases of Zhejiang Province, The First Affiliated Hospital of Wenzhou Medical University, Wenzhou, 325000, China.

Y. Guo

Hunan Provincial Key Laboratory of Advanced Materials for New Energy Storage and Conversion, School of Materials Science and Engineering, Hunan University of Science and Technology, Xiangtan 411201, China

L. Li

Department of Orthopaedics Surgery, Shandong Provincial Hospital Affiliated to Shandong First Medical University, Jinan, 250021, Shandong, China

E-mail: 13505312449@163.com (L. Li).

**
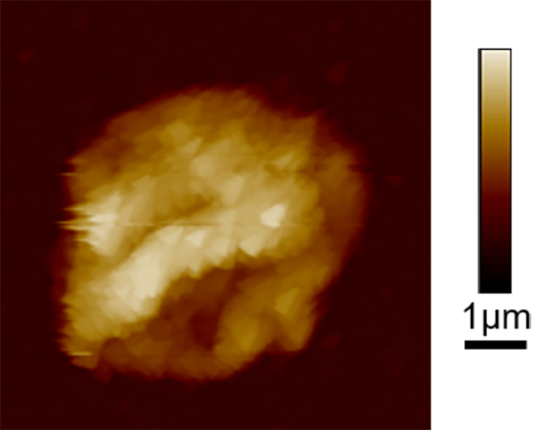
**

**Figure S1.** AFM images of PC capsule.
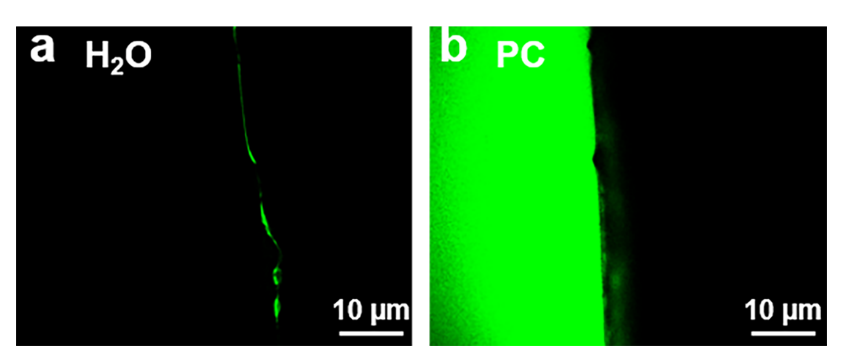


**Figure S2.** CLSM images of pure water (a) and PC solution (b).


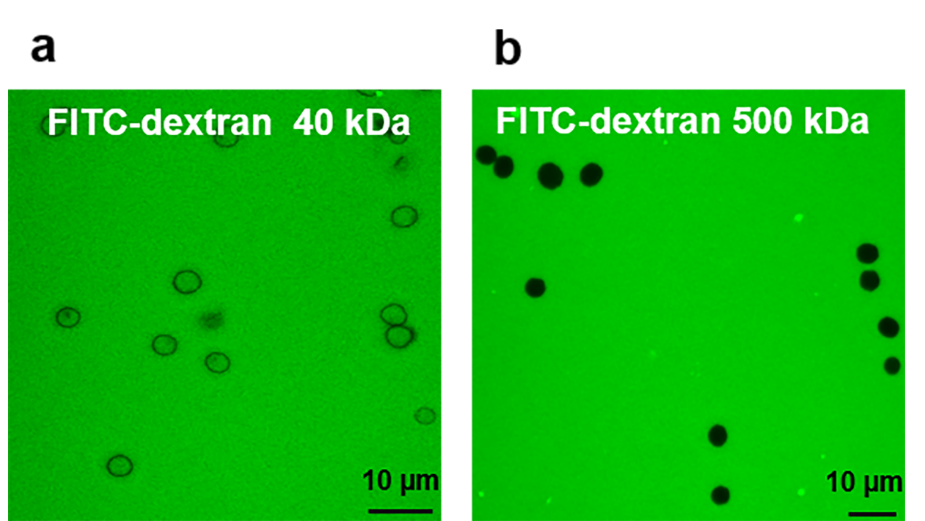


**Figure S3.** CLSM images of PC capsules against FITC dextran with Mw of 40 kDa (a) and 500 kDa (b).


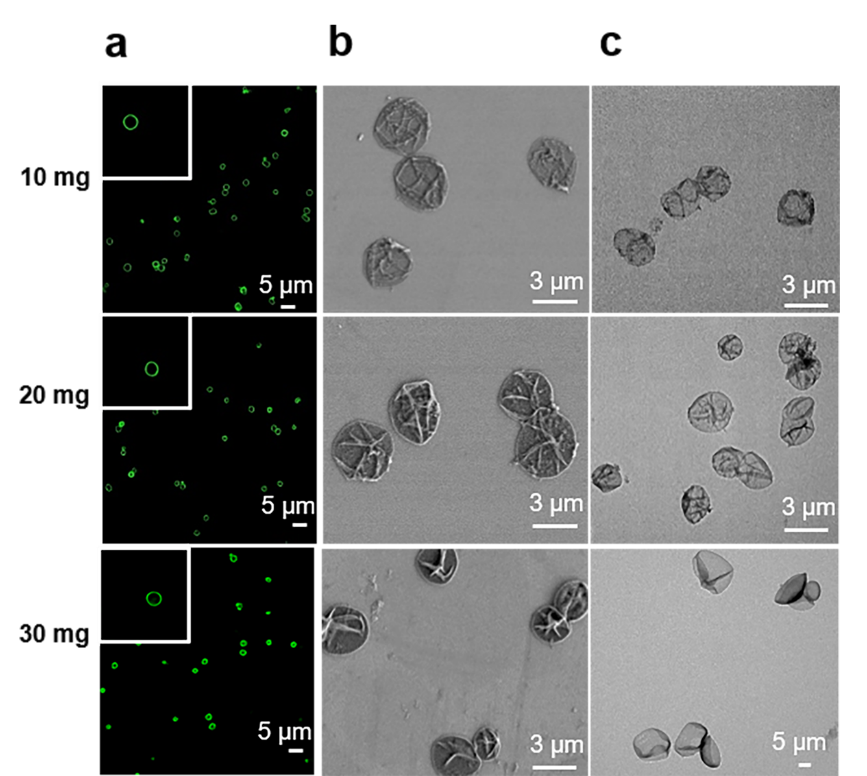


**Figure S4.** (a) SEM images, (b) TEM images, and (c) CLSM images of PC capsules which doping 10, 20, and 30 mg PC.


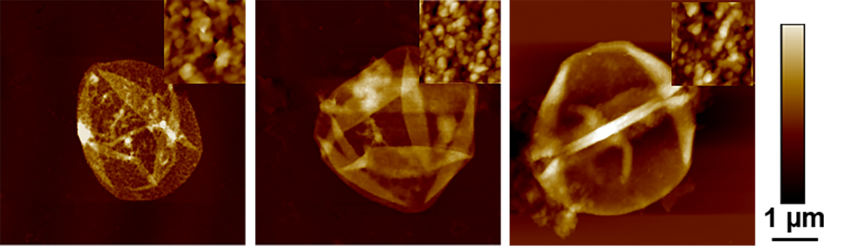


**Figure S5.** AFM images of PC capsules doping 10, 20, and 30 mg PC.


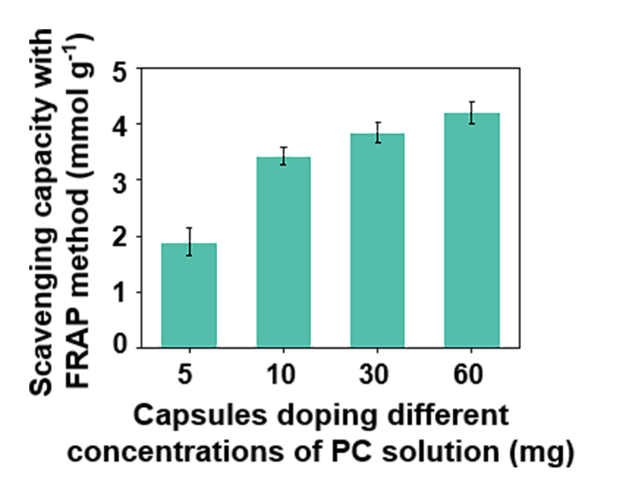


**Figure S6.** The free radical scavenging ability of PC capsules doping 10, 20, 30 and 60 mg PC. All the values are expressed as mean ± SD, n = 3.


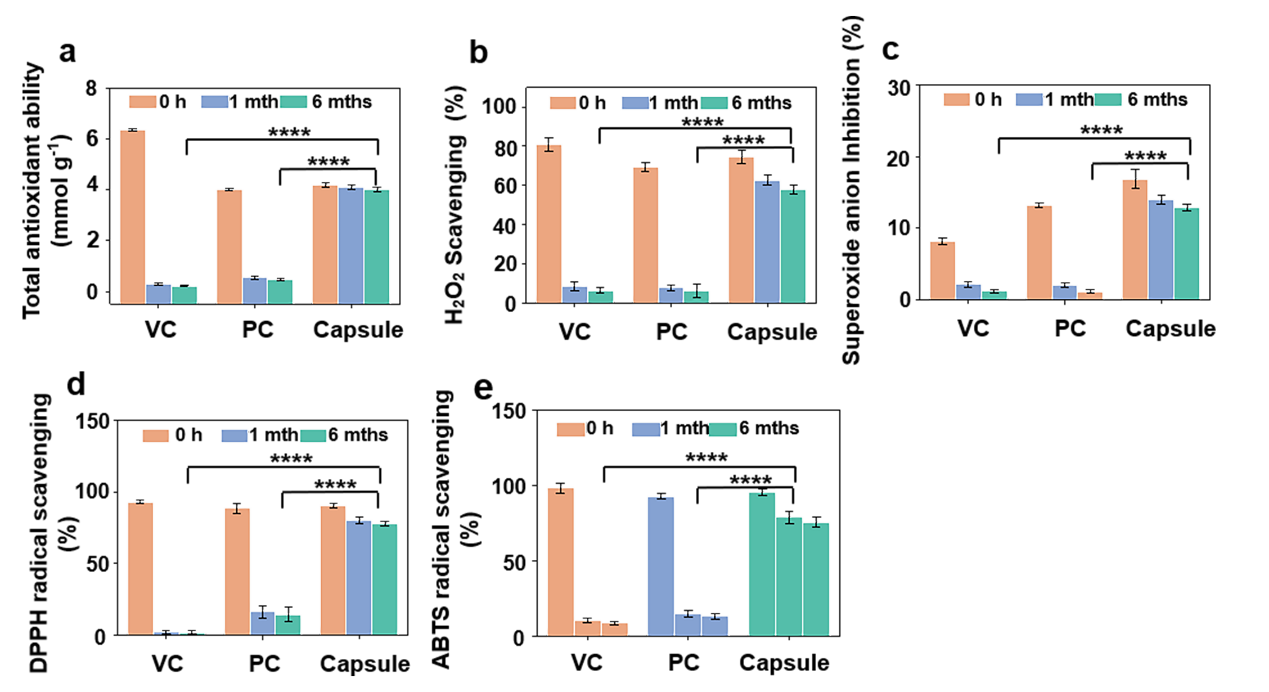


**Figure S7.** Storage in an oxidant environment for 0 h, one month and six months, (a) Total antioxidant ability, (b) H_2_O_2_, (c) O_2_^−^, (d) DPPH radical, and (e) ABTS radical scavenging ability of VC solution, PC solution, and PC capsules. The yellow column represents the effect after 0 h of placement, the blue column represents the effect after one month of placement and the green column represents the effect after six months of placement. (P values: ns P>0.5, **P < 0.01, ***P < 0.001, ****P < 0.0001, all the values are expressed as mean ± SD, n = 3).


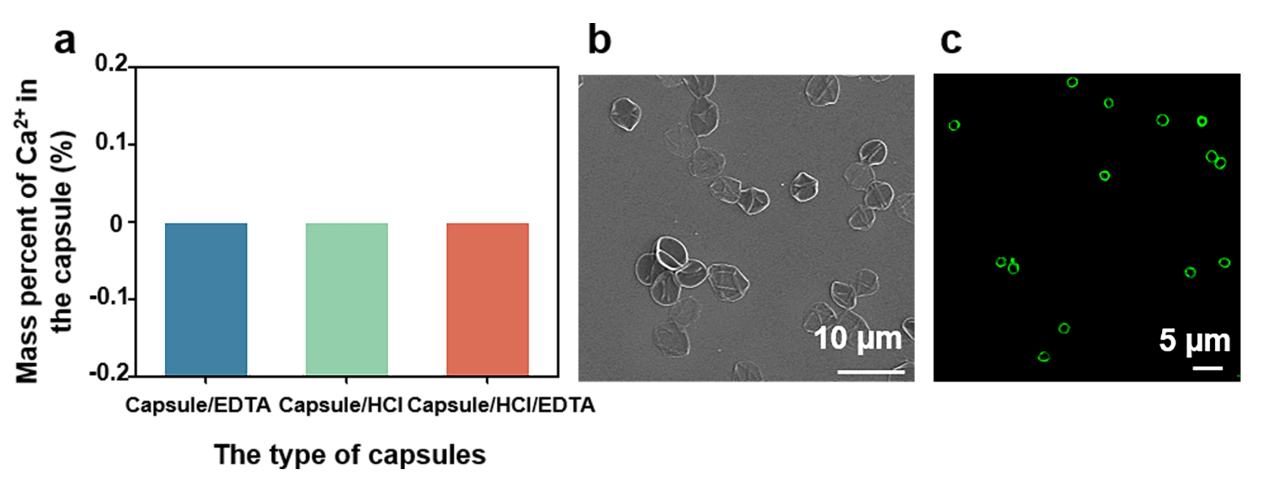


**Figure S8.** (a) The peak of Ca element from PC and PC capsules prepared by removing the template by HCl and EDTA, respectively. SEM image (b) and CLSM image (c) of PC capsules adding EDTA solution. All the values are expressed as mean ± SD, n = 3.


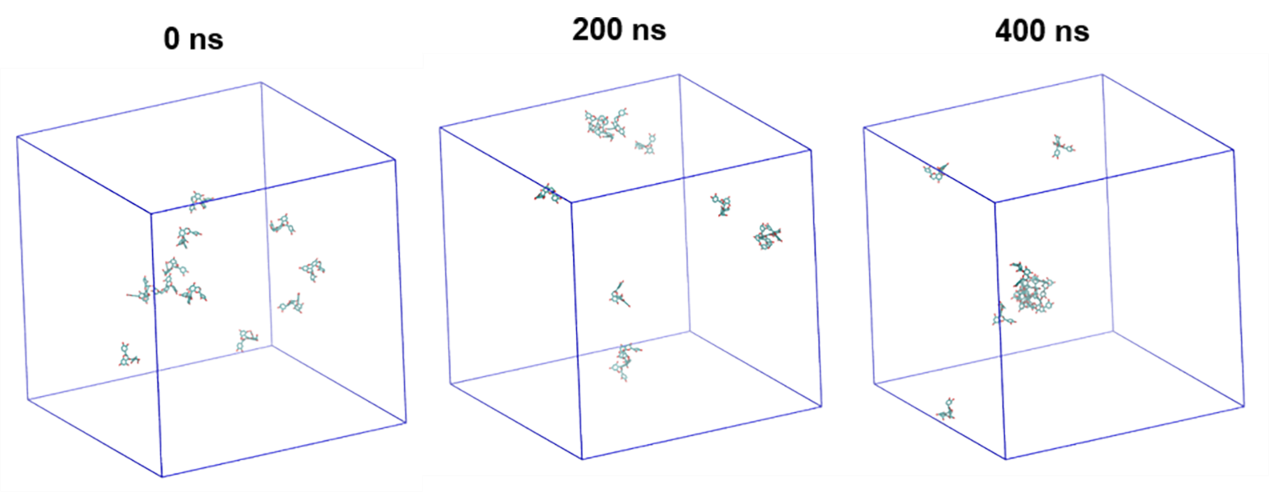


**Figure S9.** The representative snapshot (0 ns, 200 ns, 400 ns) from the all-atom molecule dynamics simulation trajectory showed the aggregation of low-density (8.5 mM L^-1^) PCD molecules.


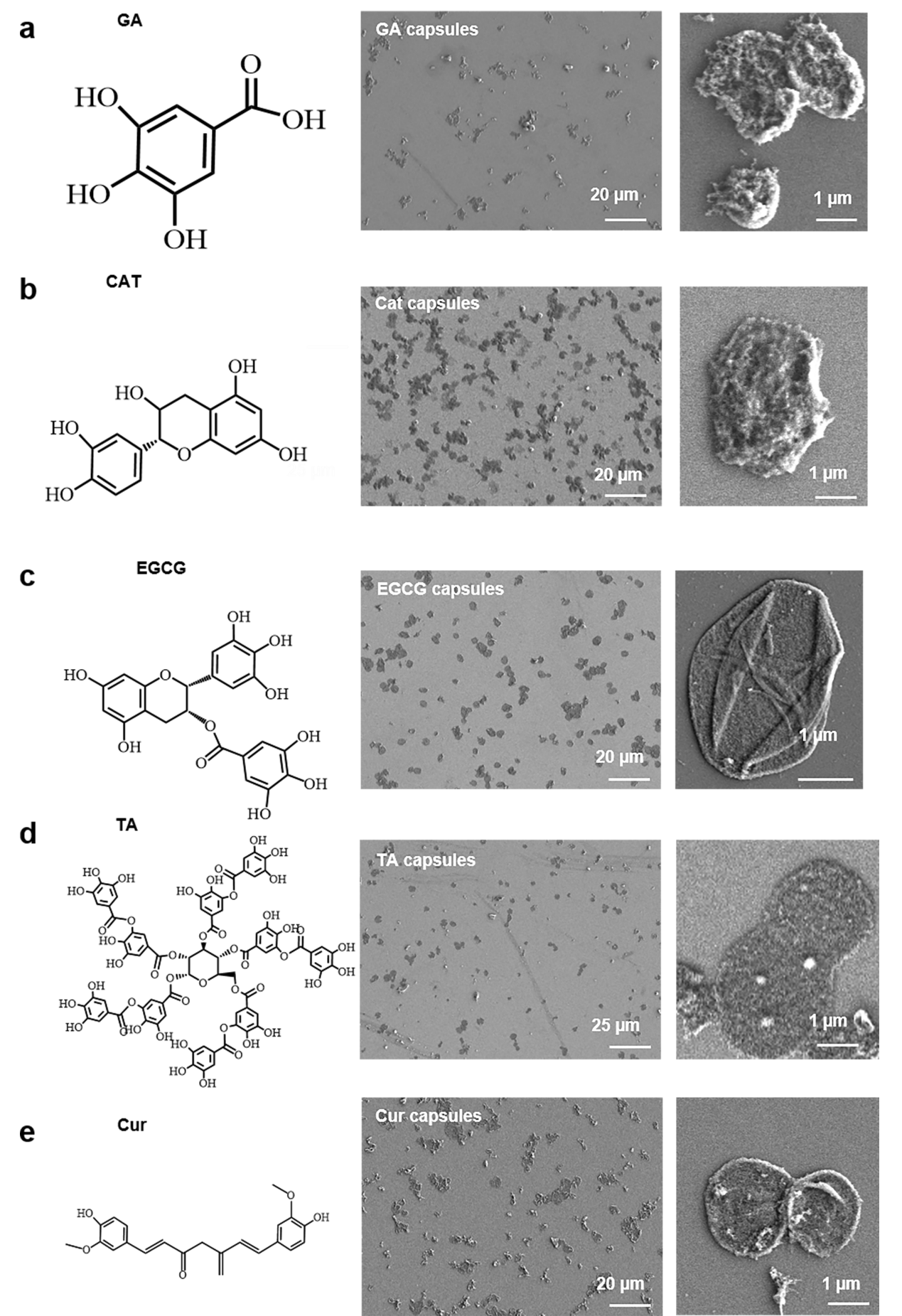


**Figure S10.** SEM images of different polyphenol capsules. (a) GA capsules. (b) Cat capsules. (c) EGCG capsules. (d) TA capsules. (e) Curcumin capsules.


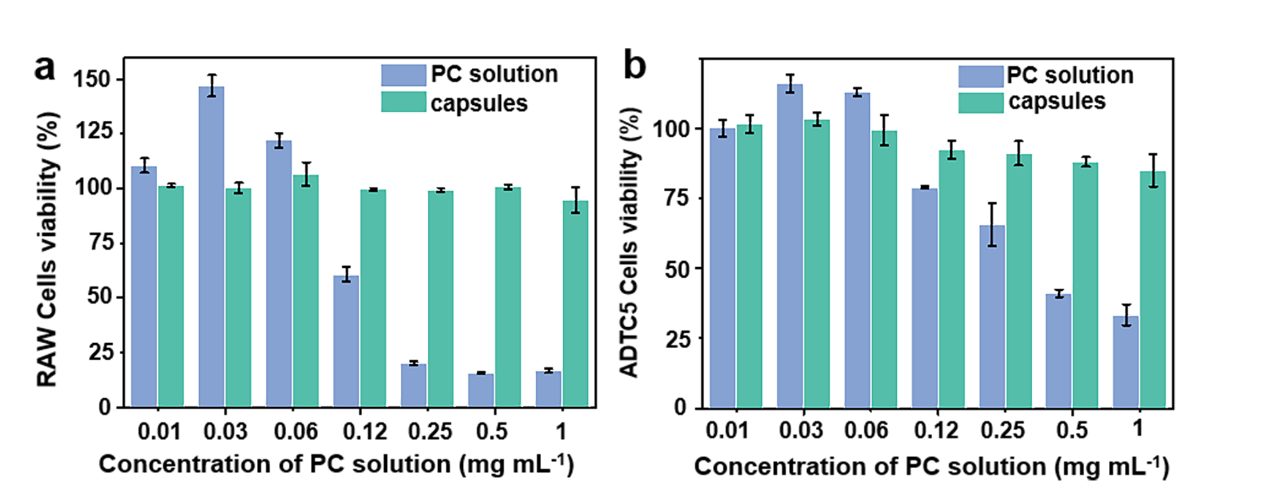


**Figure S11.** Cell viability of (a) RAW 264.7 cells and (b) ATDC5 cells after incubating with PC solution and capsules for 48 h. All the values are expressed as mean ± SD, n = 3.


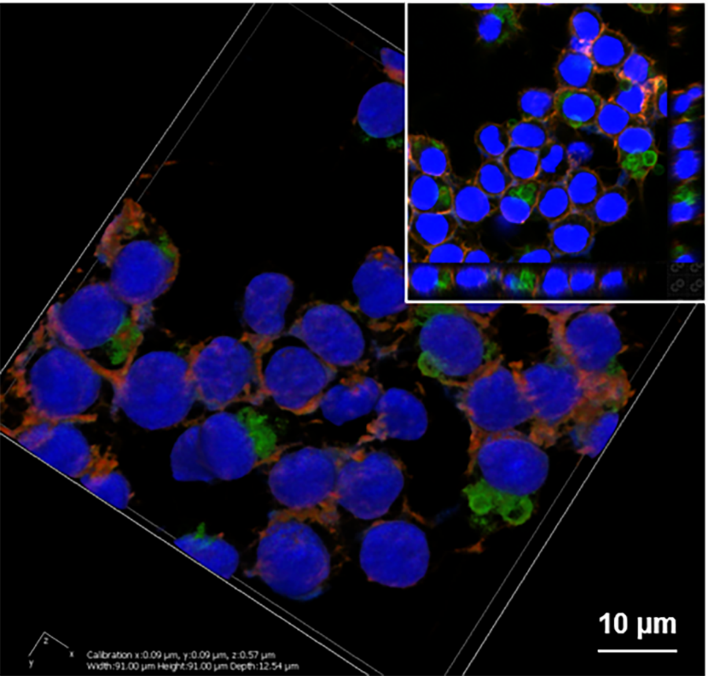


**Figure S12.** 3D-CLSM images of RAW cells after incubating with PC capsules for 24 h.


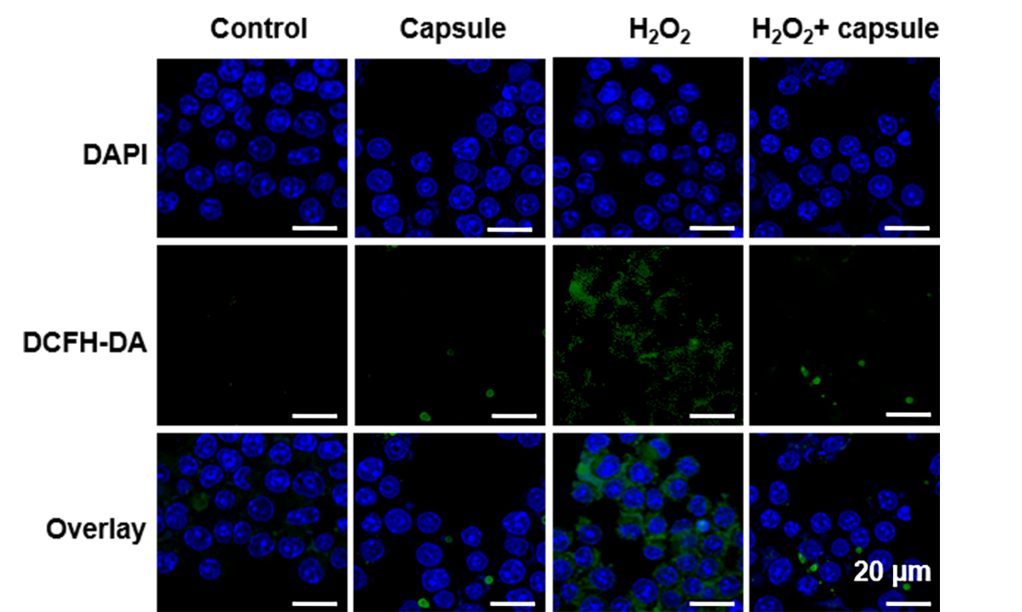


**Figure S13.** (a) The apoptosis rates of and flow cytometry. (b) The survival rate of ADTC5 induced by TUNEL fluorescent images. P values: ***P < 0.001, all the values are expressed as mean ± SD, n = 3.


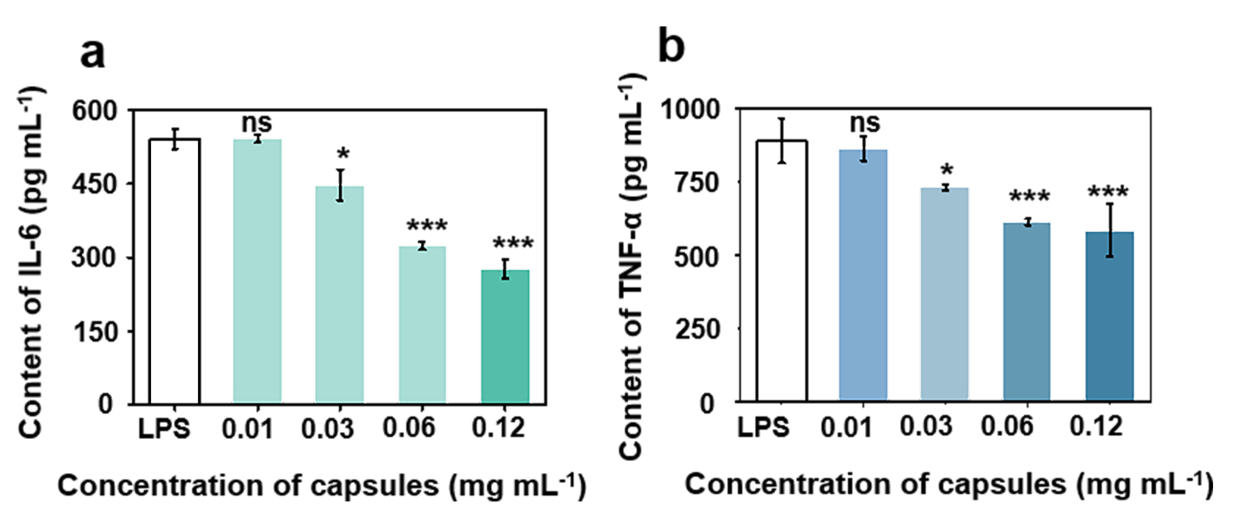


**Figure S14.** The level of IL-6 (a) and TNF-α (b) in the supernatant of RAW264.7 cells induced by LPS. P values: ns P >0.05, *P < 0.05, **P < 0.01, ***P < 0.001, all the values are expressed as mean ± SD, n = 3.

**
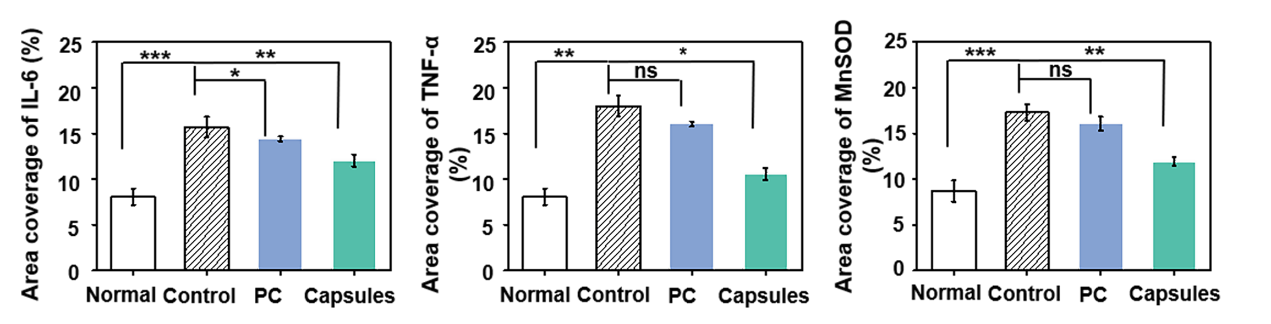
**

**Figure S15.** Representative immunohistochemical quantification of PBS, PC, and Capsules treated osteoarthritis sections stained with (a) IL-6, (b) TNF-α, and (c) MnSOD. P values: ns P >0.05, *P < 0.05, **P < 0.01, ***P < 0.001, all the values are expressed as mean ± SD, n = 3.


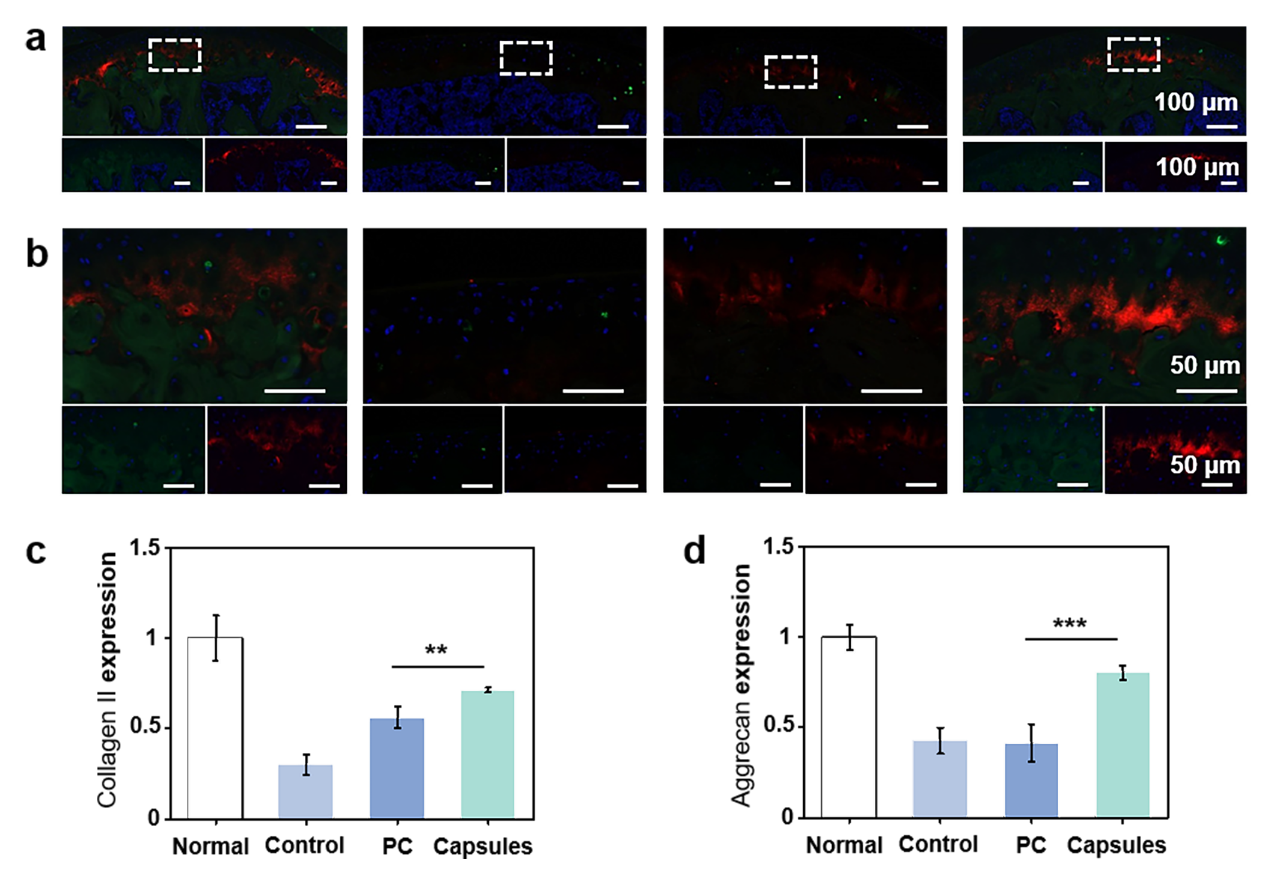


**Figure S16.** (a) The immunofluorescence staining of collagen II and aggrecan in mouse knee joint. (b) Quantitative results of collagen II and aggrecan. P values: **P < 0.01, ***P < 0.001, all the values are expressed as mean ± SD, n = 3.


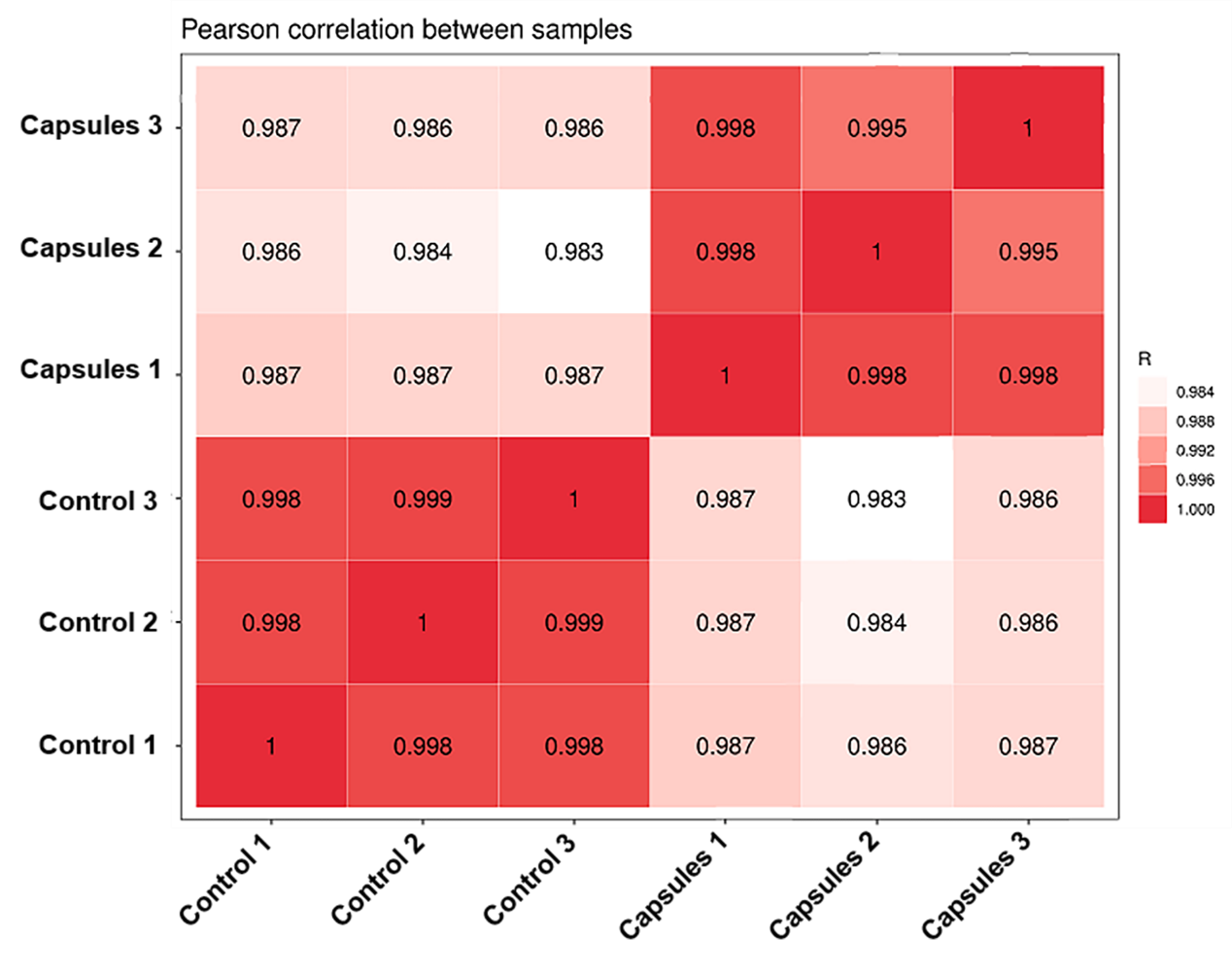


**Figure S17.** Pearson’s correlation test of osteoarthritis.


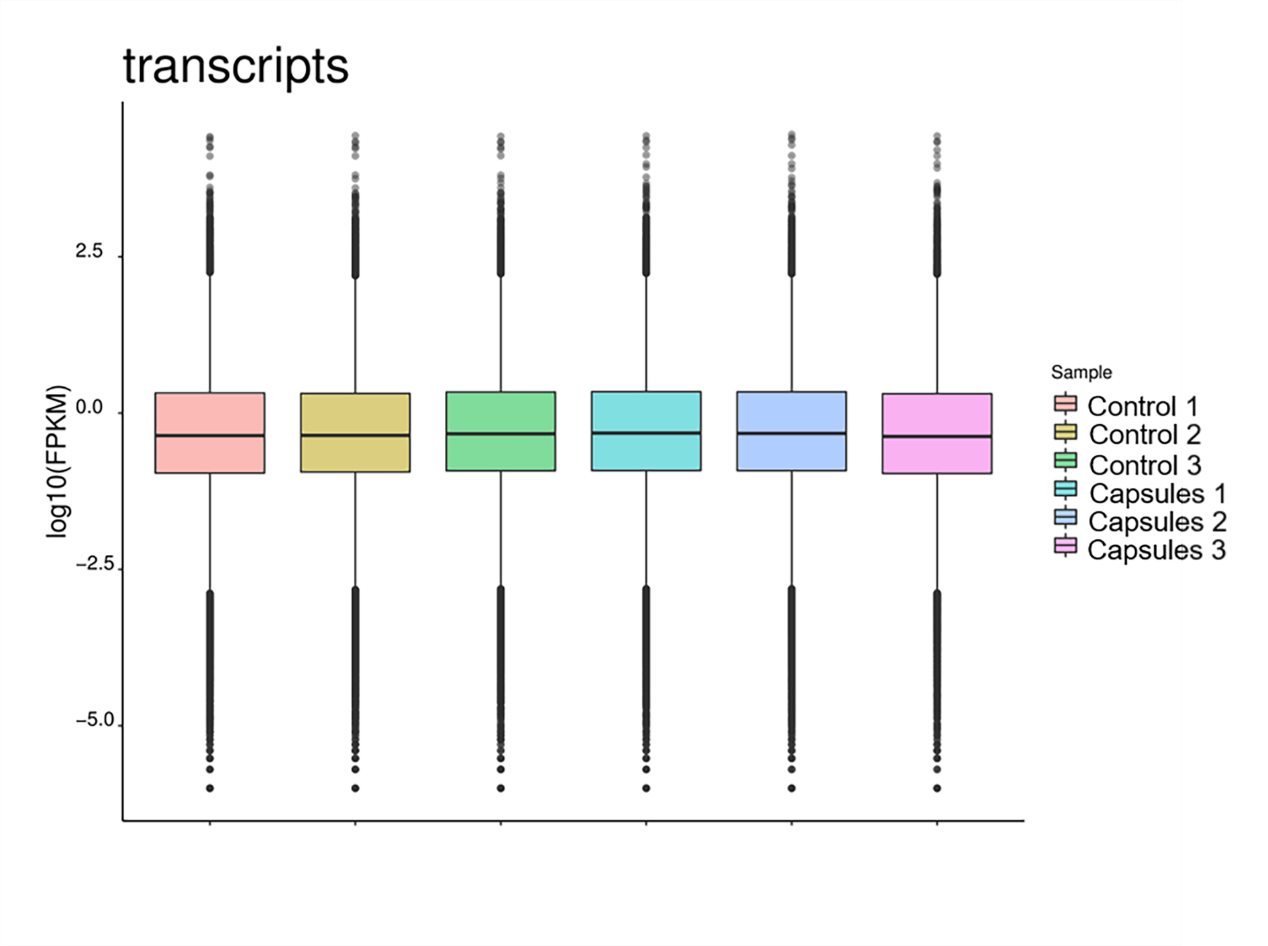


**Figure S18.** The boxplots of log10(FPKM) gene expression values of osteoarthritis.


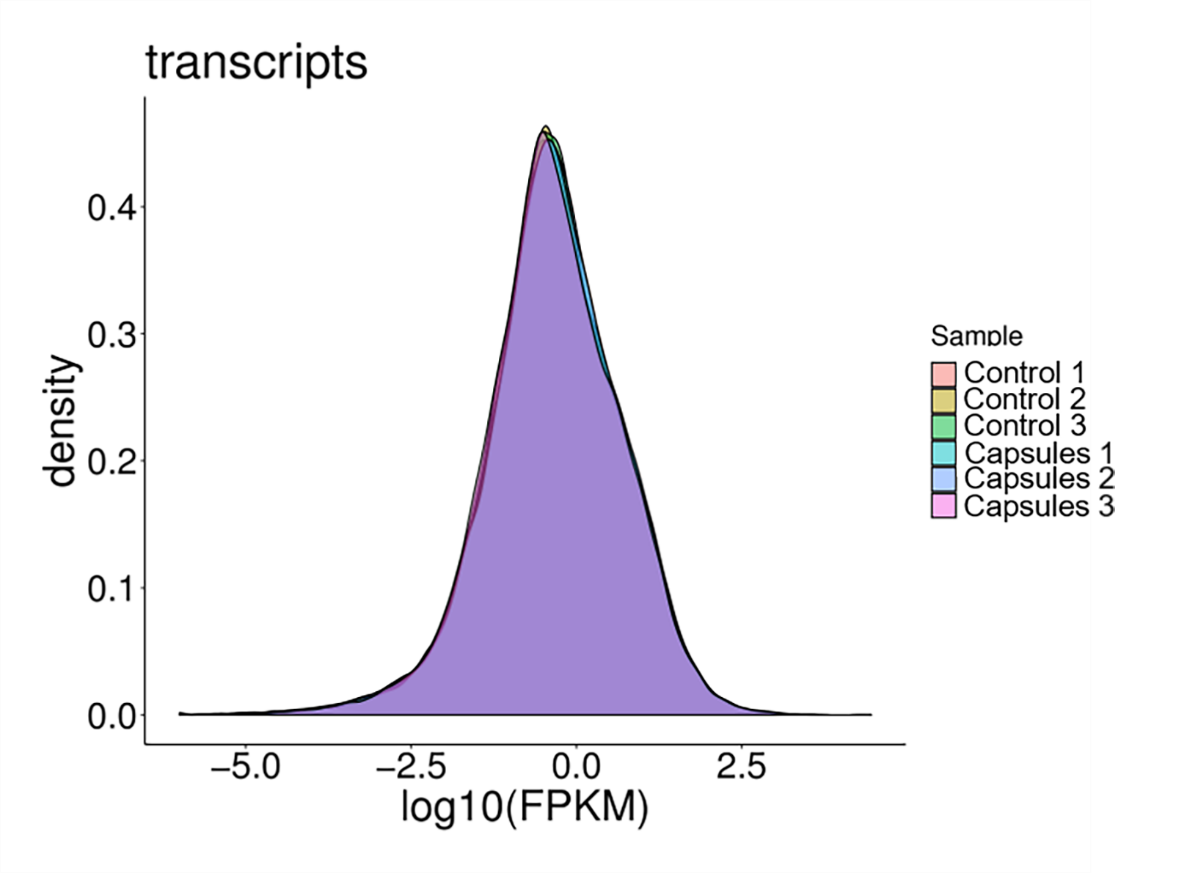


**Figure S19.** Gene expression density map of log10(FPKM) of osteoarthritis.


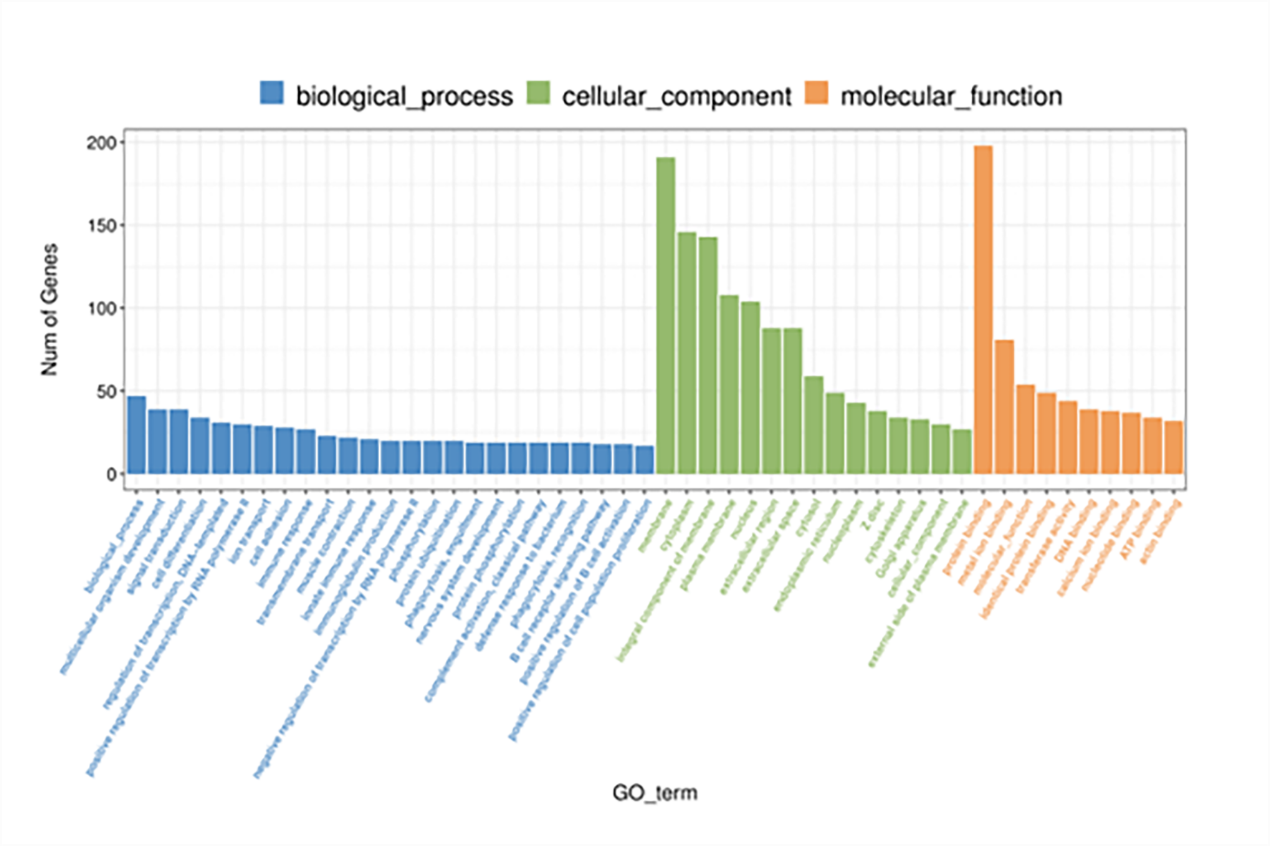


**Figure S20.** GO analysis of DEGs of osteoarthritis.


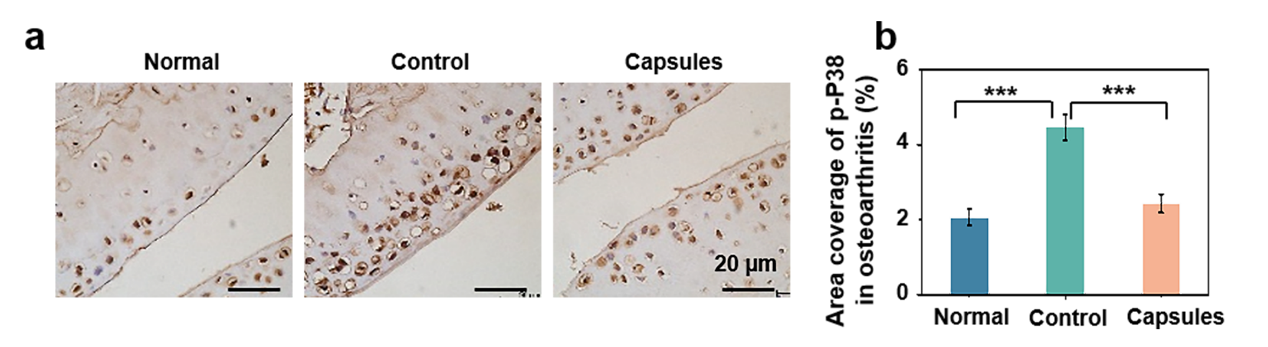


**Figure S21.** (a) Immunohistochemical staining of p-p38 in cartilage. (b) Quantitation of p-p38 in osteoarthritis. P values: ***P < 0.001, all the values are expressed as mean ± SD, n = 3.


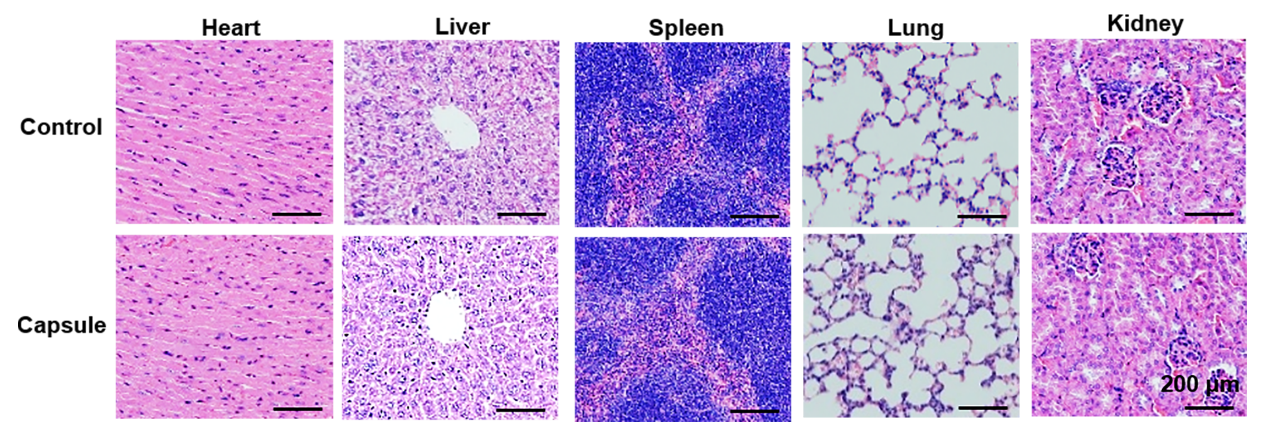


**Figure S22.** Biocompatibility assessment of capsules to major organs (heart, liver, spleen, lung, and kidney) at 14 days after administration.


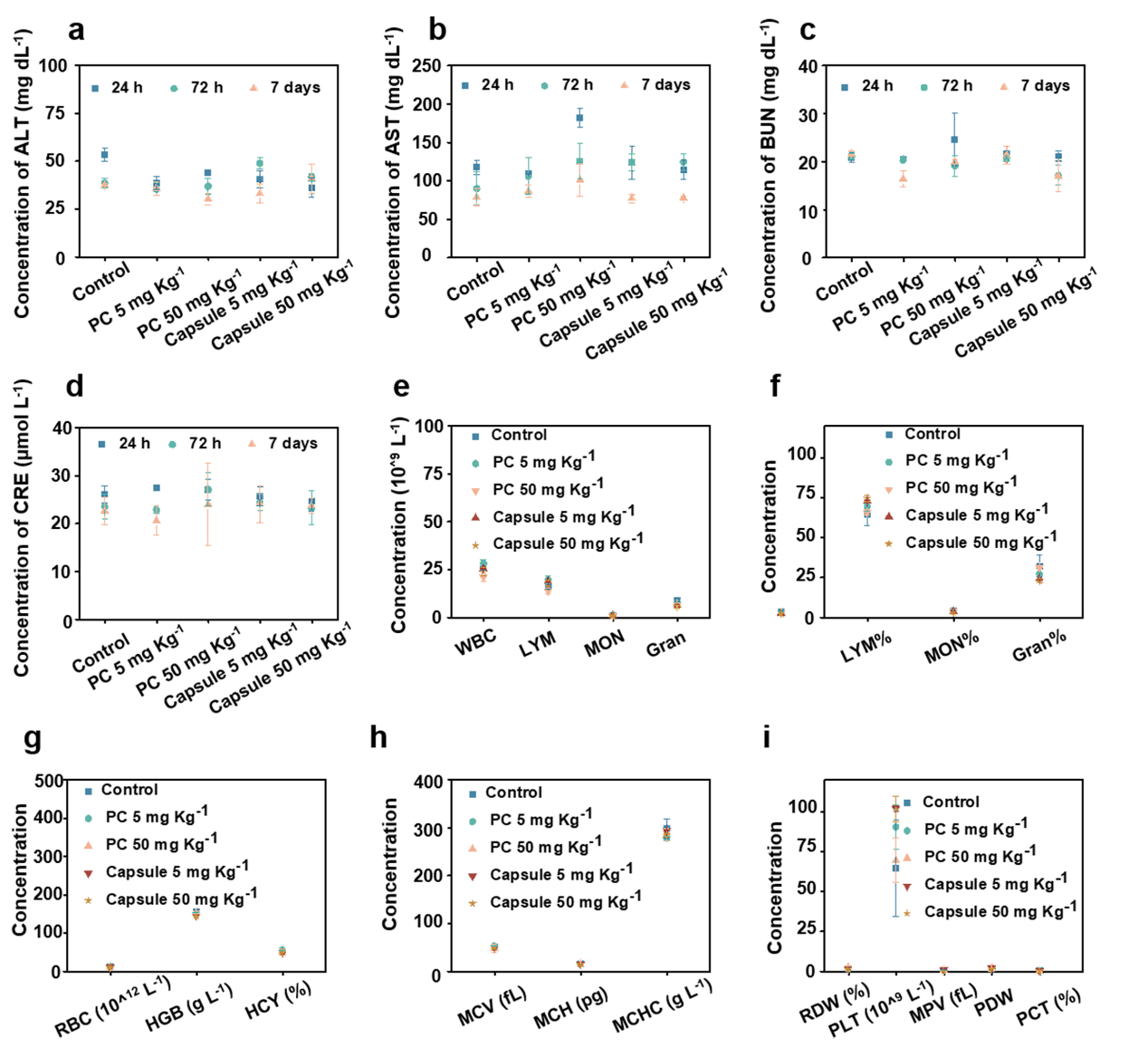


**Figure S23.** Serum levels of liver and kidney function indicators in normal mice (control) and mice intraperitoneally injected with different concentrations of PC solution and capsules after 24 h, 48 h, and 7 days: (a) aspartate transaminase (AST), (b) alanine transaminase (ALT), (c) blood urea nitrogen (BUN) and (d) creatinine (CRE). (e-i) Blood parameters in normal mice (control group) and mice intravenously after 7 days intraperitoneally injected with different concentrations of PC solution and capsules. The magnifications of all images were 200×. The scare bars in (d), (e), and (f) are 10 μm. The scare bars in (g) are 200 μm. All the values are expressed as mean ± SD, n = 3.


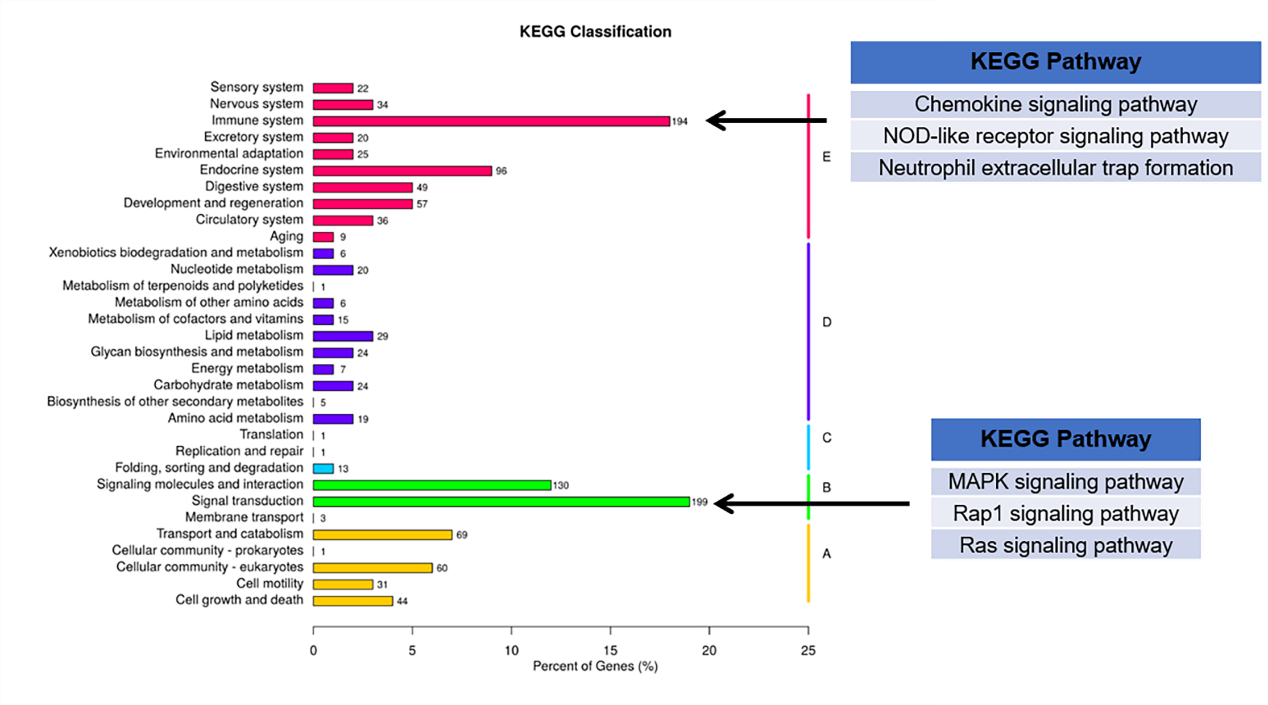


**Figure S24.** DEGs KEGG classification analysis of surgical osteoarthritis model. Bubble map of KEGG enrichment analysis of DEGs. The number of genes is indicated by the size of the dots, while the *q*-value significance is shown by the color.


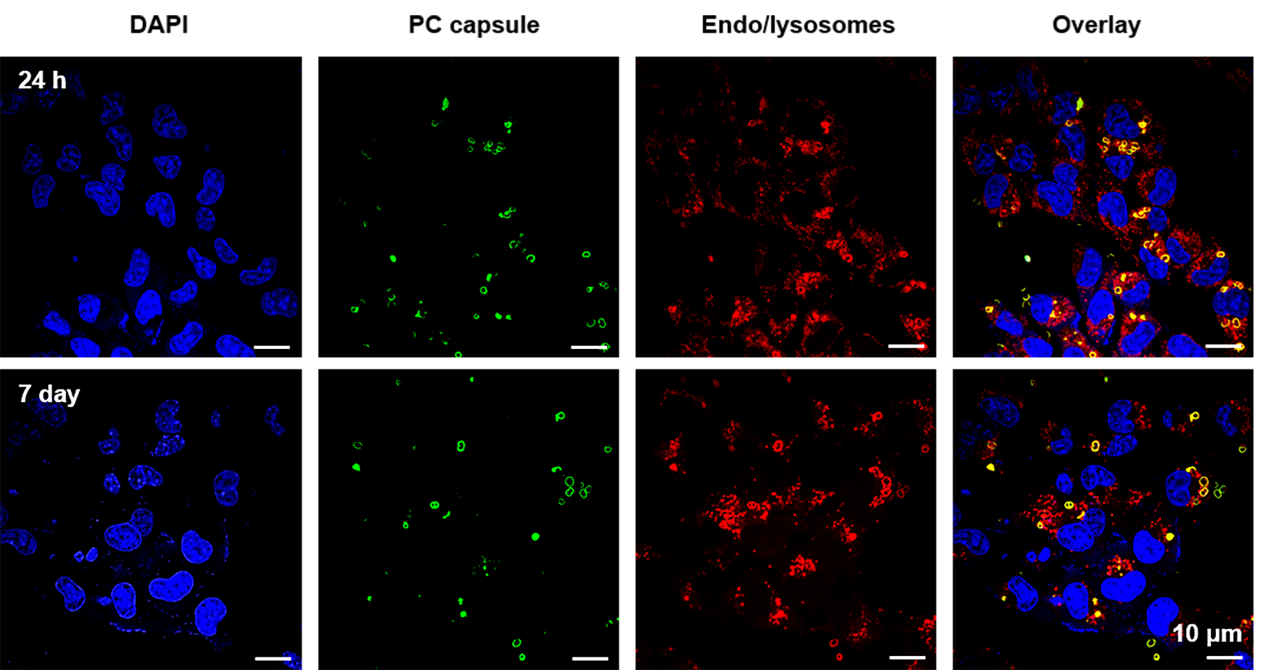


**Figure S25.** CLSM images of Lyso Tracker colocalization assay performed in ADTC5 cells after incubation for 12 h and 24 h with PC capsules. The scale bar is 10 μm.

**Table 1.** Results of generating capsules under different amounts of PC

| The amount of adding PC (mg) | 5 | 10 | 20 | 30 | 40 | 60 | 80 |
| --- | --- | --- | --- | --- | --- | --- | --- |
| Result | X | ✔ | ✔ | ✔ | ✔ | ✔ | ✘ |

“✔” means capsules can be formed, and “✘” means capsules cannot be formed.
